# Exercise dose and triglyceride reduction in postmenopausal women: a systematic review and model-based network meta-analysis

**DOI:** 10.3389/fphys.2026.1911395

**Published:** 2026-09-07

**Authors:** Junru Zeng, Hong Wang, Fengrui Shi

**Affiliations:** Wuhan Sports University, Wuhan, China

**Keywords:** dose-response relationship, exercise, network meta-analysis, postmenopausal women, triglyceride

## Abstract

**Background:**

Cardiovascular disease constitutes a leading cause of morbidity and mortality among women following menopause, with triglyceride (TG) dysregulation serving as a critical pathophysiological contributor. Despite accumulating evidence supporting the efficacy of structured exercise interventions, consensus regarding the optimal modality and “dose” required to maximize TG reduction in this demographic remains elusive.

**Methods:**

We systematically searched PubMed, Scopus, The Cochrane Library, Web of Science, CNKI, Wanfang Database, Weipu Database, and Chinese Biomedical Database through December 2025. Eligible studies comprised randomized controlled trials (RCTs) enrolling postmenopausal women, comparing aerobic exercise (AE), combined exercise (CE), resistance training (RT), high-intensity interval training (HIIT), and control (CON). A random-effects model was employed for network meta-analysis (NMA), computing mean differences (MD) and 95% credible intervals (CrI). Treatment hierarchies were established via surface under the cumulative ranking curve (SUCRA). Exercise doses were standardized to metabolic equivalent task minutes per week (MET-min/week), and a dose-response meta-analytic framework was applied to characterize potential non-linear relationships between exercise volume and TG modification.

**Results:**

51 RCTs encompassing 2,046 participants were included. Network meta-analysis demonstrated that HIIT (MD: −0.23 mmol/L, 95% CrI: −0.44 to −0.03), AE (MD: −0.20 mmol/L, 95% CrI: −0.27 to −0.13), and CE (MD: −0.17 mmol/L, 95% CrI: −0.31 to −0.03) significantly reduced TG relative to CON, whereas RT did not reach statistical significance (MD: −0.10 mmol/L, 95% CrI: −0.25 to 0.05). SUCRA rankings identified HIIT as the most efficacious intervention (79.44%), followed by AE (73.03%), CE (60.63%), and RT (34.16%), though the HIIT estimate was based on only three studies and is therefore uncertain. Dose-response modeling suggested a non-linear association between exercise volume and TG reduction, with tentative optimal therapeutic ranges: HIIT at 1,000–1,300 MET-min/week, AE at 830–1,000 MET-min/week, and CE at 830–1,200 MET-min/week, beyond which effects appeared to plateau.

**Conclusions:**

This investigation provides preliminary evidence suggesting a non-linear dose-response relationship between exercise volume and TG reduction in postmenopausal women. HIIT demonstrated the highest relative efficacy in SUCRA rankings, though this conclusion is based on limited evidence and wide credible intervals. All active interventions produced clinically meaningful TG lowering within tentative dose ranges, but the observed non-linear pattern should be interpreted cautiously given the low-to-moderate certainty of evidence. These findings challenge the simplistic “more is better” paradigm and offer hypothesis-generating guidance for precision exercise prescription, pending confirmation by future high-quality RCTs.

## Highlights

- First application of model-based network meta-analysis to investigate non-linear dose-response relationships between exercise volume and triglyceride reduction in postmenopausal women.
- High-intensity interval training showed promising efficacy in SUCRA rankings, though this conclusion is based on limited evidence and requires confirmation in larger trials.
- Non-linear dose-response patterns were suggested, with tentative optimal dose ranges for each exercise modality rather than a linear “more is better” pattern.
- Clinically meaningful triglyceride improvements were observed at moderate exercise doses, offering hypothesis-generating support for precision exercise prescription in postmenopausal dyslipidemia management.

## 1 Introduction

Cardiovascular disease (CVD) represents the predominant cause of mortality among women globally, with postmenopausal status conferring a substantially elevated risk profile compared with the premenopausal state (Song et al., 2023). The menopausal transition is characterized principally by alterations in ovarian steroidogenesis, particularly the precipitous decline in estradiol concentrations, which precipitates unfavorable modifications in lipid metabolism and body composition (Auro et al., 2014). Mounting evidence indicates that menopause is independently associated with elevations in total cholesterol, low-density lipoprotein cholesterol (LDL-C), and triglyceride (TG) concentrations, alongside impaired high-density lipoprotein cholesterol (HDL-C) functionality, collectively fostering an atherogenic lipid phenotype (Kindie et al., 2025). Among these lipid parameters, elevated TG has emerged as an independent predictor of cardiovascular risk in postmenopausal women, with recent metabolomic investigations further underscoring the pivotal role of triglyceride-rich lipoproteins in menopause-associated metabolic perturbations (Zhang et al., 2024; Zhao et al., 2024).

Physical exercise constitutes a cornerstone of non-pharmacological cardiovascular risk modification in postmenopausal women. Systematic reviews and meta-analyses have consistently demonstrated that structured exercise interventions, encompassing aerobic exercise (AE) (Luo et al., 2019), resistance training (RT) (Marques et al., 2009), and combined training (CE) (Rossi et al., 2016), exert favorable modulatory effects on the lipid profile of this population. Moreover, a recent network meta-analysis (NMA) identified combined training as the most effective intervention for TG reduction, followed by mind-body exercise (BE) and resistance training (Zhang et al., 2025). Nevertheless, despite broad consensus regarding the salutary effects of exercise on lipid parameters, substantial heterogeneity persists concerning the optimal exercise modality, intensity, and volume required to maximize TG reduction in postmenopausal women (Bernal et al., 2025).

A critical limitation of the existing evidence base is the paucity of dose-response analyses elucidating the relationship between exercise volume and TG outcomes. Although current World Health Organization (WHO) guidelines recommend a minimum of 600 MET-minutes of moderate-intensity physical activity per week for general health maintenance (Bull et al., 2020), these recommendations are predicated primarily on cardiovascular and all-cause mortality endpoints, with limited evidence specifically addressing optimal exercise dosing for TG management in postmenopausal women. Furthermore, recent dose-response investigations in metabolic syndrome patients and adolescent populations suggest that the relationship between exercise volume and lipid metabolic outcomes may be non-linear, characterized by the existence of an optimal therapeutic range. Similarly, a dose-response meta-analysis in overweight or obese adolescents identified non-linear associations between exercise intervention and improvements in the triglyceride-glucose (TyG) index and fasting glucose, with optimal effects occurring at single-session durations of 50–60 minutes and total weekly volumes of 1,000–1,500 MET-minutes, beyond which effects progressively reverted toward baseline (Yang et al., 2025). These findings suggest that the metabolic benefits of exercise on lipid parameters are confined to a defined therapeutic window, with both insufficient and excessive volumes potentially compromising efficacy—a pattern that appears generalizable across diverse age groups and metabolic states.

These observations underscore the imperative to transcend simplistic binary assessments of exercise efficacy and delineate population-specific dose thresholds for precision exercise prescription. However, conventional pairwise meta-analyses are constrained to direct comparisons and cannot synthesize indirect evidence across diverse exercise modalities and doses; conversely, conventional aggregate NMA approaches treat different doses of the same intervention as discrete entities, precluding modeling of continuous dose-response relationships. Model-based network meta-analysis (MBNMA) addresses these limitations by parameterizing dose-response functions within the NMA framework, enabling estimation of dose-response curves and identification of optimal dose thresholds (Mawdsley et al., 2016). Despite its methodological applicability for optimizing exercise prescription, no investigation has yet applied this framework to elucidate the dose-response relationship between exercise volume and TG reduction in postmenopausal women. Accordingly, this systematic review and network meta-analysis aims to: (1) compare the relative efficacy of different exercise modalities in reducing TG levels among postmenopausal women; and (2) explore the dose-response relationship between total exercise volume (weekly MET-minutes) and TG outcomes using MBNMA.

## 2 Methods

This systematic review was conducted in accordance with the Preferred Reporting Items for Systematic Reviews and Meta-Analyses (PRISMA) extension statement. The review protocol was prospectively registered with the International Prospective Register of Systematic Reviews (PROSPERO; registration number: CRD420261340607).

### 2.1 Search strategy

A comprehensive search strategy combining controlled vocabulary and free-text terms was applied across eight electronic databases: PubMed, Scopus, The Cochrane Library, Web of Science, China National Knowledge Infrastructure, Wanfang Database, Weipu Database, and Chinese Biomedical Database. No language restrictions were imposed, and the search timeframe extended from database inception through December 2025. The complete search strategy is detailed in Appendix 1.

### 2.2 Study selection

Inclusion criteria were as follows: (1) postmenopausal women as the study population; (2) intervention groups receiving any form of structured exercise training, including aerobic exercise, resistance training, high-intensity interval training, combined aerobic and resistance training, or mind-body exercise, with intervention duration ≥4 weeks; CON groups receiving usual care, placebo, or minimal lifestyle advice; (3) studies reporting triglyceride concentration changes; and (4) randomized controlled trial design.

Exclusion criteria comprised: (1) non-randomized controlled trials, quasi-experimental studies, cohort studies, case-control studies, case series, case reports, or review articles; (2) interventions involving pharmacological agents or bariatric surgery combined with exercise where the isolated effect of exercise could not be disentangled; and (3) studies failing to report TG-related outcomes or providing insufficient data for effect size computation (e.g., missing means, standard deviations, or sample sizes).

### 2.3 Data extraction

Study screening was conducted independently by two investigators (S.F.R. and Z.J.R.) in two phases. The initial screening phase evaluated eligibility based on titles and abstracts, followed by a full-text review phase for potentially eligible records. Discrepancies were resolved through consultation with a third investigator (W.H.).

### 2.4 Risk of bias and evidence assessment

Methodological quality was independently appraised by two investigators (S.F.R. and Z.J.R.) using the revised Cochrane Risk of Bias tool for randomized trials (RoB 2) (Sterne et al., 2019). The assessment framework encompassed five core domains: randomization process, deviations from intended interventions, missing outcome data, measurement of the outcome, and selection of the reported result. Based on comprehensive evaluation across these domains, each study was classified as having low risk of bias, some concerns, or high risk of bias (Higgins et al., 2011). The following information was extracted from included studies: study characteristics (design type, sample size), participant baseline data (age, BMI, health status), exercise intervention parameters (modality, intensity, frequency, duration), and outcome measurement results. To facilitate effect size computation, pre-post intervention change means and standard deviations were additionally extracted for each group. Data extraction was similarly performed independently by the aforementioned two investigators, with disagreements resolved through discussion with the third investigator (W.H.). All TG values were extracted and analyzed in mmol/L. Studies reporting TG in mg/dL were converted using the standard divisor.

### 2.5 Physical activity dose computation and dose-response analysis

Exercise intervention protocols were classified according to American College of Sports Medicine (ACSM) criteria, with classification performed independently by two investigators (Thompson et al., 2013). Weekly exercise doses for each intervention group were standardized to MET·min/week using the formula: Dose = MET value × weekly frequency × single-session duration (min). For multi-component exercise programs, total dose was computed as the sum of constituent components. MET value assignment rules were as follows: (1) for studies explicitly reporting exercise speed or modality, corresponding MET values were obtained from the 2024 Adult Compendium of Physical Activities (Herrmann et al., 2024); (2) for studies describing intensity ranges only, midpoint values from ACSM absolute intensity thresholds were applied: light 2.5 MET, moderate 4.5 MET, vigorous 8.0 MET; (3) HIIT was classified as vigorous intensity by default; (4) resistance training intensity was determined based on percentage of one-repetition maximum (%1RM) or rating of perceived exertion (RPE) scale, with corresponding Compendium MET values (light/moderate 3.5 MET, vigorous 6.0 MET, circuit training 8.0 MET). Missing data were addressed through email correspondence with original authors. Detailed MET assignments for each intervention group, including intensity categorization rationale and calculation steps, are provided in **Supplementary Appendix 2**.

We acknowledge that MET-based standardization introduces measurement error, particularly for HIIT (where work-rest ratios, peak intensities, and total active time vary substantially across protocols) and RT (where load, sets, repetitions, rest intervals, and muscle groups targeted are critical physiological determinants not fully captured by MET values). Readers should interpret the proposed dose windows as approximate ranges derived from available evidence rather than precise prescription thresholds.

Effect sizes were computed using pre-post change values (means and standard deviations). For exercise modalities with ≥3 included studies, pairwise meta-analysis was conducted using a random-effects model, with mean difference (MD) as the effect size metric and 95% credible intervals (CrI) reported. Heterogeneity was assessed via the I² statistic (I² > 50% considered substantial), and publication bias was evaluated using Egger’s test and adjusted funnel plots (Yang et al., 2023).

Following PRISMA-NMA guidelines, a Bayesian random-effects NMA integrating direct and indirect evidence was performed. Network geometry was constructed to illustrate comparison relationships among exercise modalities. The consistency model was fitted using the gemtc package in R software, with a normal likelihood function for the outcome and MD of baseline changes as the effect size. Markov Chain Monte Carlo (MCMC) parameters: 4 chains, 20,000 iterations, 5,000 burn-in. Heterogeneity was expressed as the posterior median of τ with 95% CrI. Global inconsistency was assessed by comparing deviance information criterion (DIC) values between consistency and unrelated mean effects (UME) models (ΔDIC < 5 indicating no significant inconsistency); local inconsistency was evaluated using the node-splitting method (p < 0.05 indicating inconsistency). The probability of each intervention being the optimal treatment and SUCRA values were computed, with forest plots and league tables generated (Spineli, 2025).

### 2.6 Network meta-analysis of dose-response

The MBNMA framework was applied to evaluate dose-response relationships between exercise dose and TG changes under a Bayesian random-effects model (Pedder et al., 2019). Prior to analysis, key assumptions including network connectivity (**Supplementary Figures 9**, **S10**), consistency, and transitivity were confirmed as satisfied (**Supplementary Table 6**) (White et al., 2012). An initial “split” NMA treating each dose as an independent intervention was performed to guide function form selection, followed by comparison of Emax models, restricted cubic splines (3 knots), non-parametric monotonic models, and quadratic models with common and random treatment effects (Noma et al., 2020). The optimal model was selected based on DIC, residual deviance, and other goodness-of-fit indices. Minimum effective doses were estimated using 95% prediction intervals, and intervention-dose combinations were ranked according to improvement probability (Crippa and Orsini, 2016). Effect sizes with 95% CrI excluding zero were considered statistically significant. All analyses were performed in R (version 4.5.1) using the MBNMAdose package (Neupane et al., 2014).

Network meta-regression was conducted to explore potential moderating effects of age, body mass index, intervention duration, exercise frequency, and publication year on effect sizes. Sex proportion was not included as a covariate because all included studies exclusively enrolled postmenopausal women.

## 3 Results

### 3.1 Characteristics of included studies

**Figure 1:**
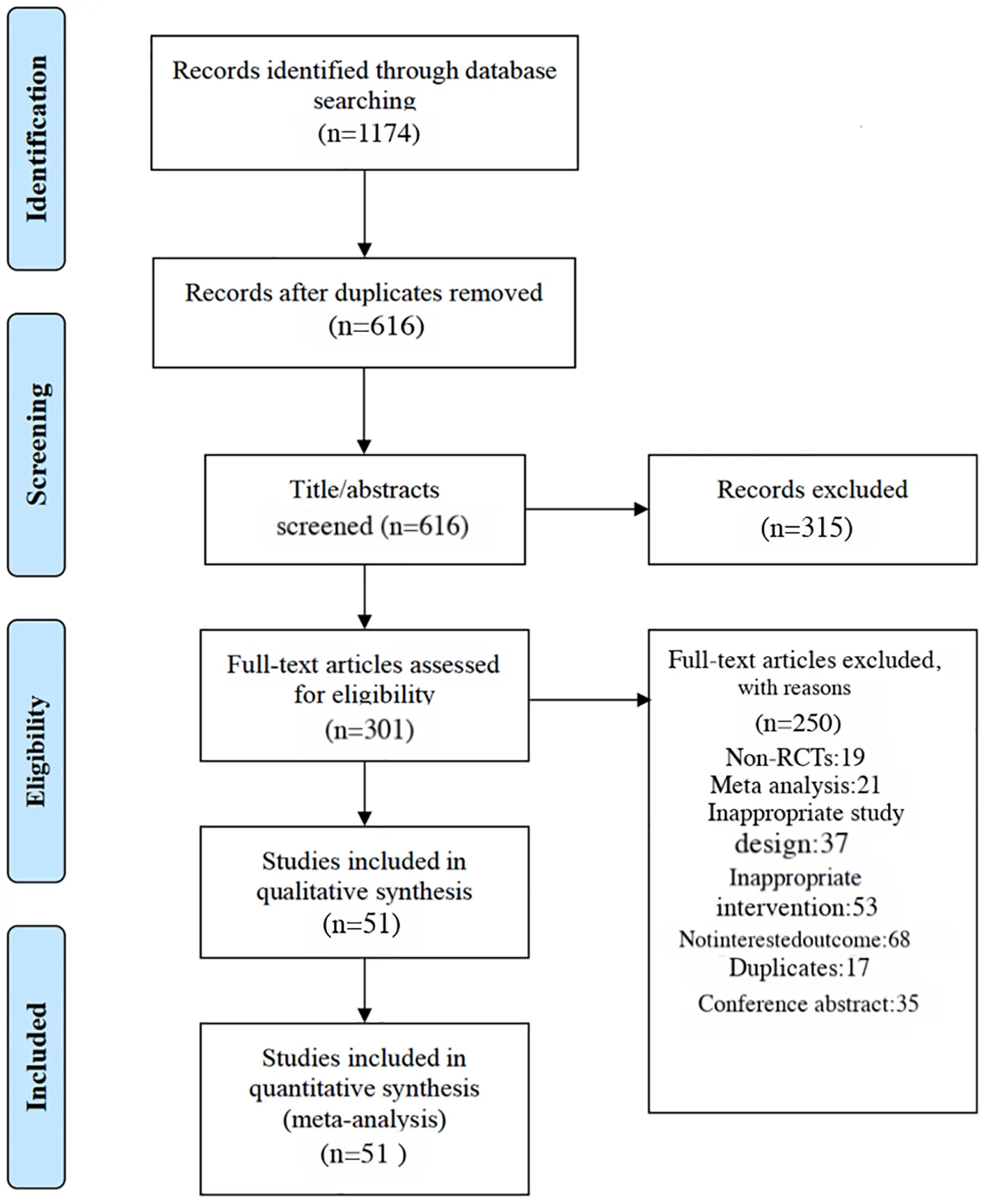
Flow diagram.

Systematic searching identified 1,174 potentially relevant records. After deduplication, 616 articles remained for title and abstract screening, of which 315 were excluded and 301 were assessed for full-text eligibility. Of these, 250 were excluded for reasons detailed in **Figure 1**: 19 non-RCTs, 21 meta-analyses, 37 studies with inappropriate study designs, 53 with inappropriate interventions, 68 with outcomes not of interest, 17 duplicate reports, and 35 conference abstracts. Consequently, 51 studies were included in the review and meta-analysis. The complete screening and selection process is illustrated in **Figure 1**.

### 3.2 Basic characteristics of included studies

51 RCTs comprising 2,046 participants were included, encompassing four exercise intervention categories: AE (39 studies), RT (12 studies), CE (12 studies), and HIIT (3 studies).

### 3.3 Risk of bias and evidence assessment

**Figure 2:**
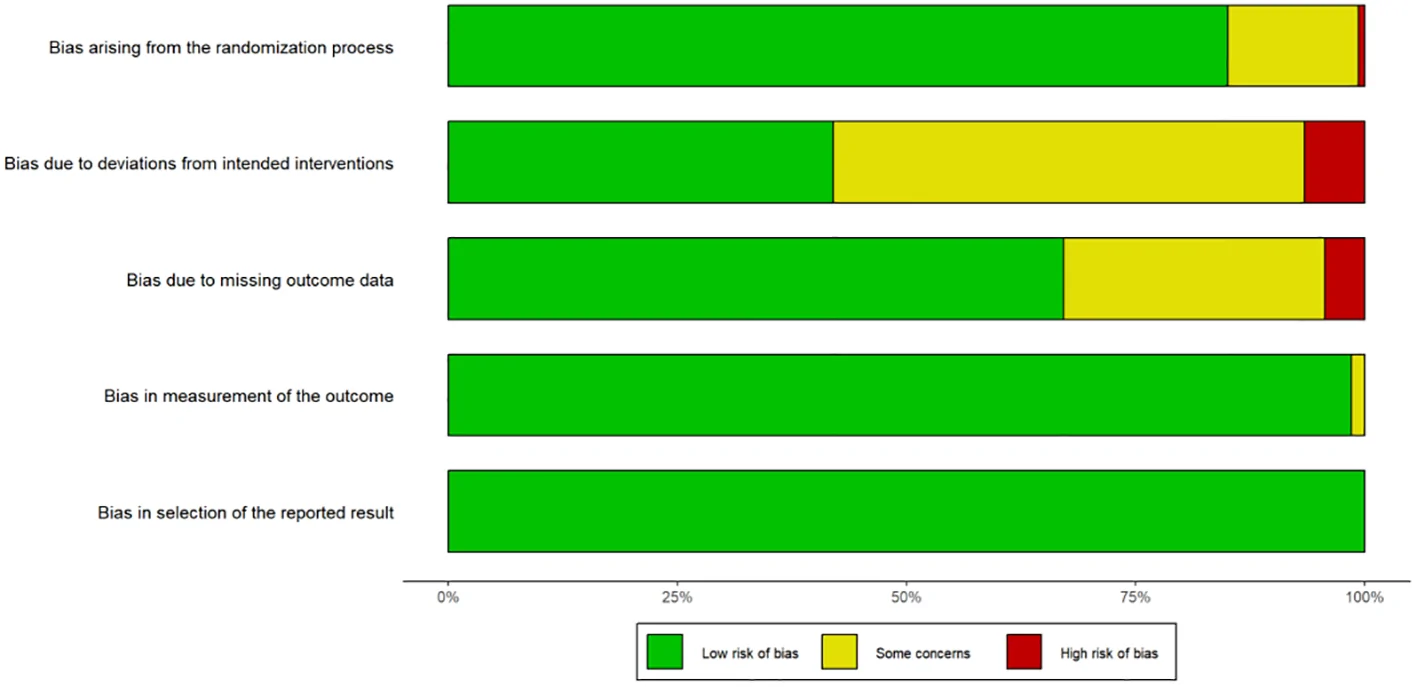
Risk of bias.

Among the 51 included studies, 8 were rated as high risk of bias, 33 as having some concerns, and 10 as low risk of bias (**Figure 2**); domain-specific and overall risk of bias assessments for individual studies are detailed in **Supplementary Appendix 3**. CINeMA evaluation of network comparison evidence confidence rated most comparisons as “very low” to “moderate” certainty; summaries are provided in **Supplementary Appendix 4**.

### 3.4 Pairwise meta-analysis

Pairwise meta-analysis was conducted for exercise categories with ≥3 included studies. Pooled results demonstrated that compared with CON, AE (MD: −0.20mmol/L, 95% CrI: −0.27 to −0.13), CE (MD: −0.28mmol/L, 95% CrI: −0.48 to −0.08), and RT (MD: −0.10mmol/L, 95% CrI: −0.25 to 0.05) exhibited TG-lowering effects, although RT failed to achieve statistical significance. Significant heterogeneity was observed across comparisons (I² > 50%). Egger’s test and adjusted funnel plots suggested potential publication bias (**Supplementary Appendix 5**).

### 3.5 Network meta-analysis

#### 3.5.1 Network structure and model fit

The network geometry illustrated comparison relationships among four exercise interventions and control (**Supplementary Figure 1**). Node size reflected sample size, and line thickness represented the number of direct comparison studies. The network demonstrated good connectivity, with AE serving as the primary common comparator linking most interventions.

Model fit diagnostics supported the superiority of the random-effects model over the fixed-effects model (DIC: 157.1 vs. 308.8; residual deviance: 87.9 vs. 258.8). The between-study standard deviation was estimated at 0.14 (95% CrI: 0.09, 0.20). The unrelated mean effects (UME) model produced comparable DIC values (160.1), with ΔDIC < 5 relative to the consistency model, indicating no significant inconsistency (**Supplementary Table 2**).

#### 3.5.2 Consistency assessment

Node-splitting analysis revealed no significant inconsistency at the 0.05 level for any treatment comparison (**Supplementary Table 3**). All p-values exceeded 0.05, and credible intervals for direct and indirect estimates overlapped, supporting the transitivity assumption required for network meta-analysis.

#### 3.5.3 Relative treatment effects

Relative effect league tables and corresponding forest plots are presented in **Supplementary Table 4** and **Supplementary Figure 2**, respectively. Compared with CON, all active exercise interventions demonstrated TG-lowering effects: HIIT (MD: −0.23mmol/L, 95% CrI: −0.44 to −0.03), AE (MD: −0.20mmol/L, 95% CrI: −0.27 to −0.13), CE (MD: −0.17mmol/L, 95% CrI: −0.31 to −0.03), and RT (MD: −0.10mmol/L, 95% CrI: −0.25 to 0.05). Notably, the RT effect estimate did not exclude the null value. In head-to-head comparisons, HIIT appeared superior to RT (MD: −0.13mmol/L, 95% CrI: −0.35 to 0.08), though this difference did not reach statistical significance. No meaningful differences were detected between AE and CE (MD: 0.03mmol/L, 95% CrI: −0.12 to 0.17) or between AE and HIIT (MD: −0.03mmol/L, 95% CrI: −0.24 to 0.17).

#### 3.5.4 SUCRA rankings

Cumulative ranking probabilities and SUCRA values are presented in **Supplementary Table 5** and **Supplementary Figure 3**. HIIT achieved the highest SUCRA value (79.44%), followed by AE (73.03%), CE (60.63%), RT (34.16%), and CON (2.75%). These rankings suggest that HIIT may carry the highest probability of being the optimal intervention for reducing TG in postmenopausal women, but this conclusion is tentative given the limited number of trials and wide credible intervals.

### 3.6 Dose-response network meta-analysis

#### 3.6.1 Model assumptions and network connectivity

Network connectivity was verified at both treatment and dose levels, with no evidence of network fragmentation (**Supplementary Figures 9**, **S10**). Consistency assessment comparing consistency and UME models demonstrated good consistency (DIC: −91.2 vs. −93.2; pD: 69.6 vs. 69.7), supporting the validity of indirect comparisons (**Supplementary Table 9**). Transitivity was further assessed via MBNMA node-splitting at the treatment-dose level; with the exception of AE_1000 vs. Placebo_0 (p = 0.029), no significant inconsistency was detected, and the direct and indirect estimates for this comparison were directionally consistent (**Supplementary Table 10**; **Supplementary Figure 11**).

#### 3.6.2 Model selection

An initial “split” network meta-analysis treating each dose as an independent intervention was performed to guide function form selection (**Supplementary Figures 12**, **S13**). Multiple dose-response functions were subsequently compared, including Emax models, restricted cubic splines (3 knots), non-parametric monotonic models, and quadratic (second-order polynomial) models with common and random treatment effects (**Supplementary Table 11**). The quadratic model with random treatment effects demonstrated optimal fit (DIC: −88.9; residual deviance: 88.725; SD: 0.137, 95% CrI: 0.09–0.20) and was selected for all subsequent analyses. Deviance plots confirmed adequate model fit, with the majority of data points contributing <1.5 to posterior mean deviance (**Supplementary Figures 14**, **S15**). Fit plots further validated that the quadratic model appropriately captured observed dose-response patterns across different exercise modalities and dose levels (**Supplementary Figures 16**, **S17**).

#### 3.6.3 Overall dose-response relationship

**Figure 3:**
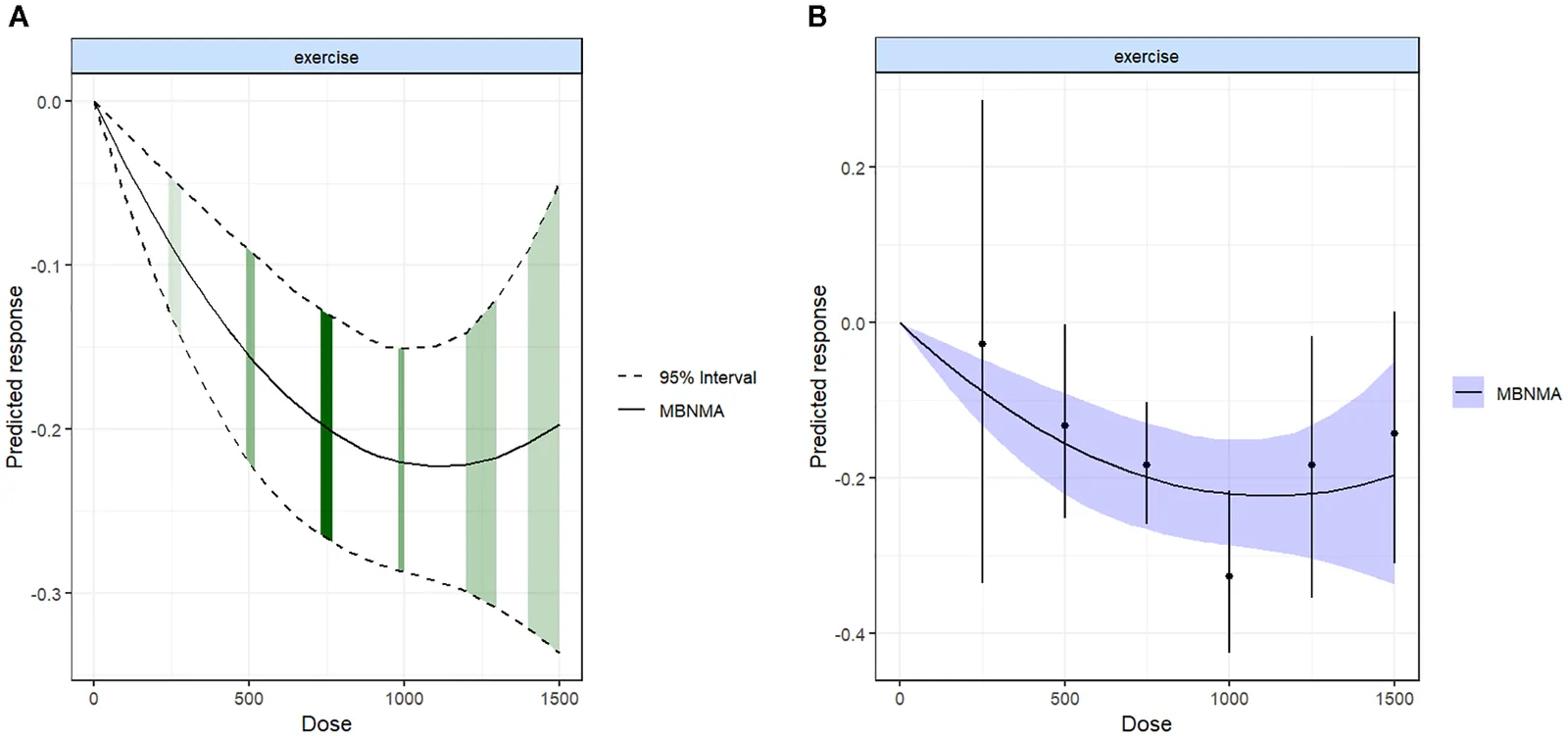
Dose-response relationship between exercise dose and triglyceride change. **(A)** Overall dose-response model with prediction interval and observed data. **(B)** Model-based dose-response curve with observed effect estimates.

The overall dose-response relationship between exercise dose (MET-min/week) and TG change is illustrated in **Figure 3a**. The quadratic model suggested a non-linear relationship characterized by an initial decline in TG levels with increasing exercise dose, followed by attenuation and plateauing at higher doses. The steepest decline occurred in the low-to-moderate dose range (approximately 250–1,000 MET-min/week) in **Figure 3b**. Beyond approximately 1,000–1,200 MET-min/week, the magnitude of TG reduction diminished, suggesting a plateau effect rather than a clear reversal. The 95% prediction interval indicated that the potential therapeutic window for TG reduction likely resides within the 500–1,200 MET-min/week range, with uncertain effects beyond this threshold due to sparse data.

#### 3.6.4 Dose-response relationships by intervention

**Figure 4:**
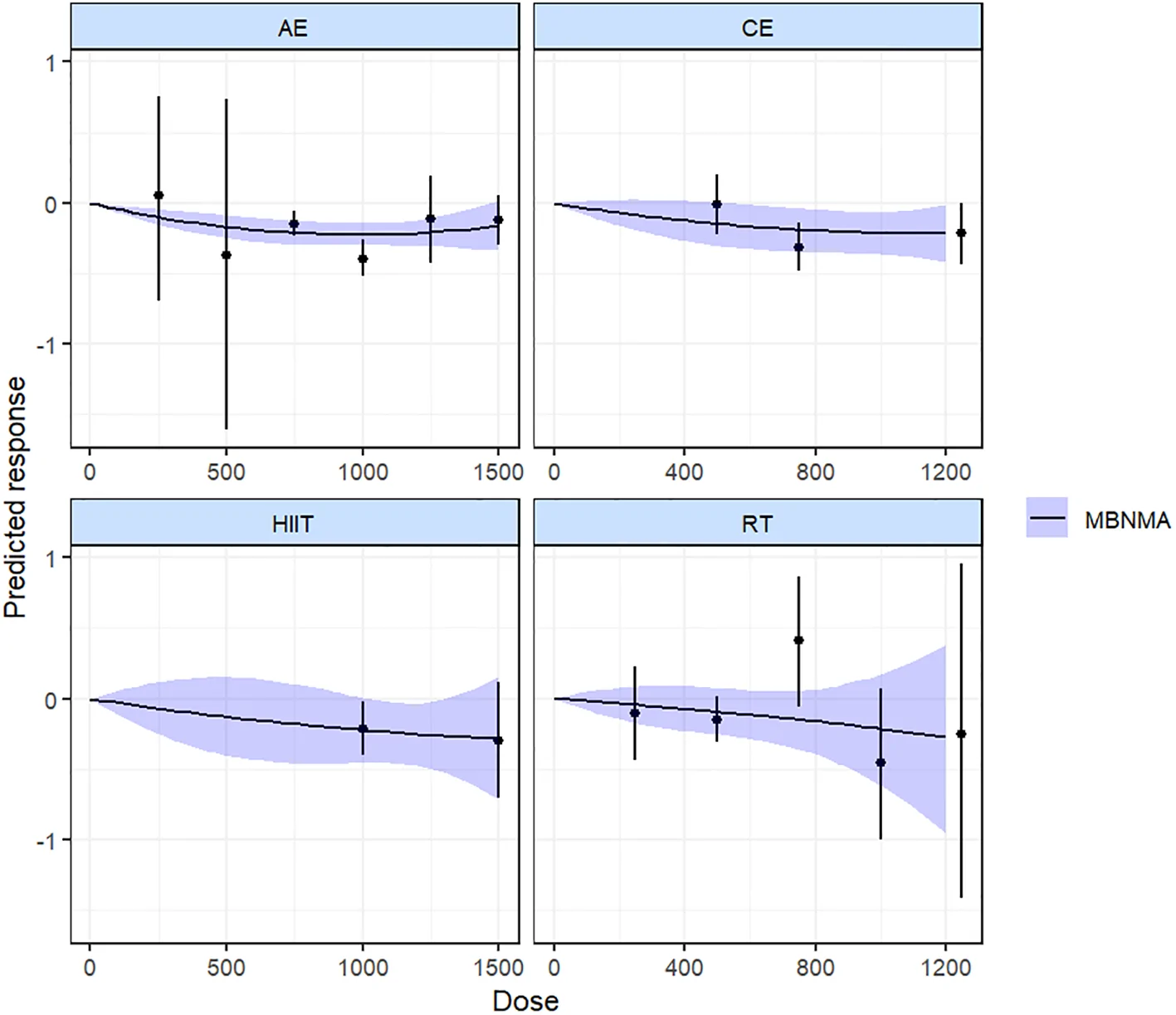
Exercise modality-specific dose-response relationships for AE, CE, HIIT, and RT.

Intervention-specific dose-response curves are presented in **Figure 4**. Distinct non-linear patterns were observed across exercise modalities: Aerobic Exercise (AE): AE exhibited a pronounced dose-response relationship, with significant TG reduction occurring at moderate doses (approximately 500–1,000 MET-min/week). The curve peaked at approximately 830–1,000 MET-min/week, with attenuation observed at higher doses (>1,200 MET-min/week). The shaded density plot indicated relatively robust data support within the 500–1,500 MET-min/week range. High-Intensity Interval Training (HIIT): HIIT displayed the most favorable dose-response profile among all interventions, with the steepest initial decline and sustained efficacy across a broader dose range. Tentative optimal effects were estimated at 1,000–1,300 MET-min/week, with maintained benefits even at higher doses. However, the evidence base for HIIT was relatively sparse. Combined Exercise (CE): CE demonstrated a moderate dose-response relationship, with optimal effects in the 830–1,200 MET-min/week range. The curve was relatively flat compared with AE and HIIT, suggesting more consistent but less pronounced dose-dependent effects. Resistance Training (RT): RT exhibited the shallowest dose-response slope among all active interventions. While modest TG reduction was observed across dose ranges, effects were weaker and more variable, with wider credible intervals reflecting greater uncertainty.

#### 3.6.5 Efficacy ranking by dose

**Figure 5:**
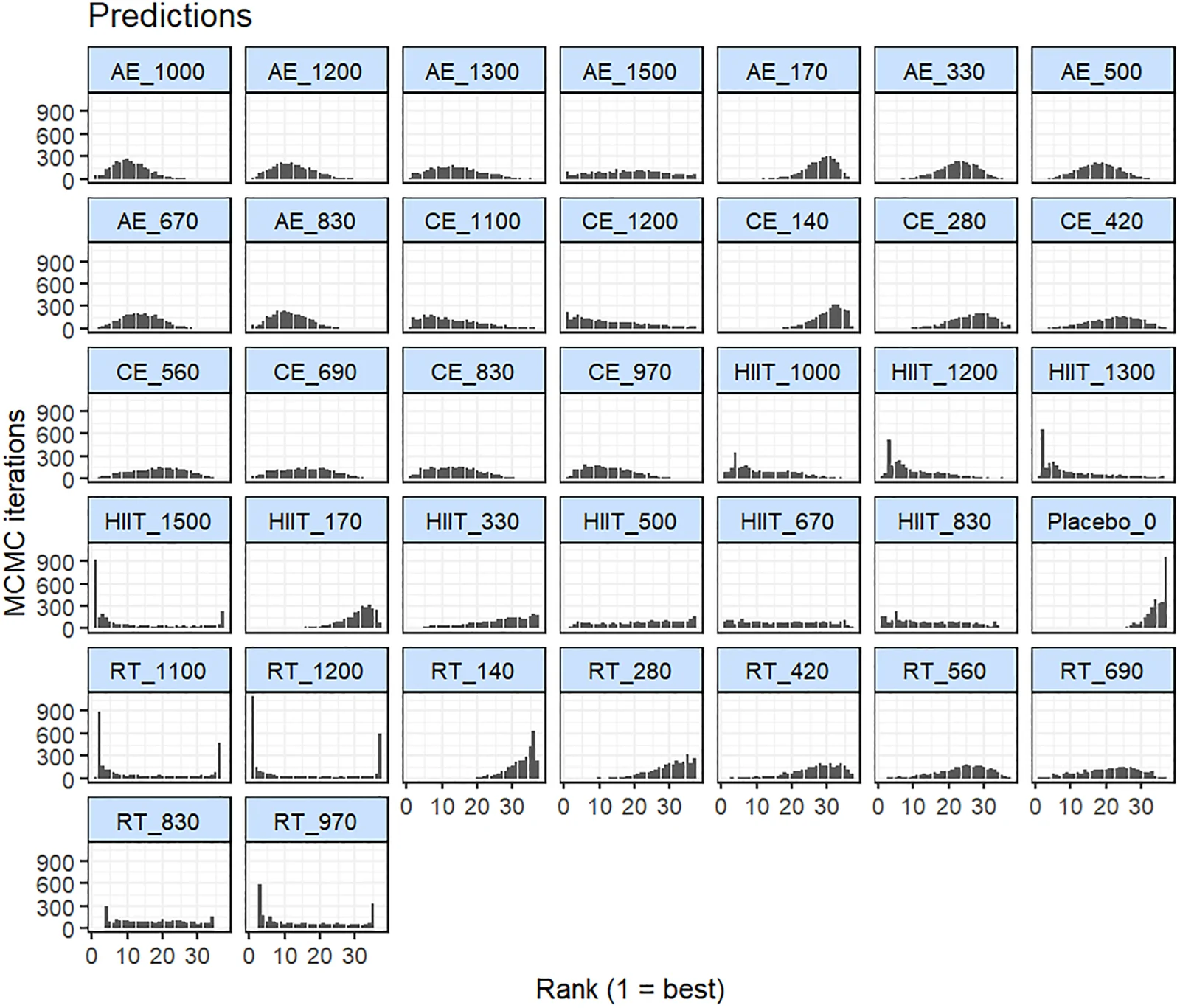
Shows the probability of each intervention to be ranked from best to worst (estimated after up to 4000 iterations).

Probability-based ranking of intervention-dose combinations is illustrated in **Figure 5** and **Supplementary Table 12**. HIIT at 1,200 MET-min/week achieved the highest probability of optimal ranking, though this estimate is highly uncertain given the sparse evidence base (mean rank: 10.55; median: 8), closely followed by AE at 1,000 MET-min/week (mean rank: 10.63; median: 10) and HIIT at 1,300 MET-min/week (mean rank: 10.66; median: 7). Notably, several moderate-dose combinations outperformed their higher-dose counterparts: AE at 830 MET-min/week (mean rank: 11.29) ranked higher than AE at 1,500 MET-min/week (mean rank: 18.63), and CE at 970 MET-min/week (mean rank: 12.68) outperformed CE at 1,400 MET-min/week. Conversely, the lowest-ranked active interventions were concentrated at both extremes of the dose spectrum—very low doses (e.g., AE_170, RT_140, CE_140) and very high doses (e.g., AE_1500, HIIT_1500)—further reinforcing the non-linear nature of the dose-response relationship. CON (labeled ‘Placebo_0’ in the MBNMA model) consistently occupied the lowest ranking (mean: 34.23; median: 35).

### 3.7 Sensitivity analysis

Sensitivity analyses were conducted to assess result robustness. Following exclusion of high risk of bias studies (retaining 74 data points), model fit indices remained comparable (random-effects model DIC: 116.8 vs. 157.1 for the full dataset), between-study heterogeneity decreased (SD: 0.07, 95% CrI: 0.01–0.15), and relative treatment rankings remained qualitatively unchanged. Similarly, exclusion of small studies (<20 participants) yielded consistent results (random-effects model DIC: 116.8; SD: 0.07), supporting the stability of primary findings (**Supplementary Table 5**).

### 3.8 Meta-regression

Network meta-regression was performed to explore potential effect modifiers. No statistically significant associations were identified for age, BMI, intervention duration, exercise frequency, or publication year (all p > 0.05), suggesting that observed treatment effects were robust across these covariates. However, the limited number of studies reporting complete covariate data may have constrained statistical power to detect moderate moderating effects.

## 4 Discussion

### 4.1 Summary of principal findings

This systematic review and network meta-analysis, encompassing 51 RCTs and 2,046 postmenopausal women, systematically elucidated the dose-response relationships between different exercise modalities and their respective doses and TG reduction within a model-based network meta-analytic framework. The principal findings can be summarized at three levels: First, among all exercise interventions, high-intensity interval training (HIIT) demonstrated the highest SUCRA ranking for TG reduction (79.44%), followed by aerobic exercise (AE, 73.03%) and combined exercise (CE, 60.63%), with resistance training (RT) showing comparatively limited efficacy (34.16%). However, the HIIT estimate was derived from only three studies with wide credible intervals, rendering this ranking highly uncertain. Second, a significant non-linear quadratic relationship was suggested between exercise dose and TG change, exhibiting an inverted U-shaped curve characteristic with tentative optimal therapeutic windows rather than simple linear increments. Third, tentative dose intervals were estimated across exercise modalities: HIIT at 1,000–1,300 MET-min/week, AE at 830–1,000 MET-min/week, and CE at 830–1,200 MET-min/week, beyond which effects plateaued or attenuated. These findings offer hypothesis-generating insights that may inform future precision exercise prescription research, pending validation by larger, high-quality RCTs.

### 4.2 Relative efficacy of different exercise modalities

#### 4.2.1 Superiority of HIIT and its physiological basis

The highest SUCRA ranking observed for HIIT in this investigation suggests potentially greater efficacy for TG reduction; however, this finding should be interpreted cautiously because it is based on only three included studies with wide credible intervals.. However, we emphasize that this finding is based on only three included studies, and the credible intervals for HIIT comparisons were wide. Although existing data support its optimal ranking, additional high-quality RCTs are warranted before HIIT can be confidently recommended as the preferred intervention for TG reduction in this population. The superiority of HIIT can be elucidated through multiple metabolic mechanisms. First, HIIT activates the peroxisome proliferator-activated receptor gamma coactivator 1-alpha (PGC-1α) signaling pathway, promoting skeletal muscle mitochondrial biogenesis and enhancing fatty acid oxidation capacity—an effect particularly critical in the context of postmenopausal estrogen deficiency (Vannuchi and Pisani, 2025). Second, the excess post-exercise oxygen consumption (EPOC) effect induced by HIIT extends the post-exercise period of active lipid metabolism, enhancing triglyceride clearance (Cheng et al., 2024). Third, high-intensity intermittent stimuli more effectively activate hormone-sensitive lipase (HSL), promoting adipose tissue lipolysis and recycling of circulating free fatty acids (Naves et al., 2018). It is noteworthy that the evidence base for HIIT in this study was relatively limited (only 3 studies); although existing data support its optimal ranking, additional high-quality RCTs are warranted to consolidate this conclusion.

#### 4.2.2 Robust efficacy and dose window of aerobic exercise

AE, as the most extensively investigated intervention category (39 studies), demonstrated robust TG-lowering effects in this study. Its mechanisms primarily involve sustained energy expenditure-induced negative energy balance, suppression of hepatic very-low-density lipoprotein (VLDL) synthesis, and enhancement of lipoprotein lipase (LPL) activity (Liu et al., 2024). A study specifically looking at how different amounts of exercise affect blood lipids in postmenopausal women showed that when exercise reaches around 700–1500 MET·minutes per week, the decrease in triglycerides tends to level off or hit its maximum (Carpeggiani Weber et al., 2025). These results suggest that the exercise threshold required for lipid benefits in postmenopausal women may be lower than the volume recommended for overall health maintenance, providing important evidence for the clinical translation of the “minimum effective dose” concept.

Clinical relevance of TG reductions: The average TG reduction of approximately −0.20 mmol/L observed for AE corresponds to roughly 15–18 mg/dL. While modest in absolute terms, such reductions are clinically meaningful in the context of atherogenic dyslipidemia, particularly among postmenopausal women with elevated baseline TG (≥1.7 mmol/L or 150 mg/dL). However, we acknowledge that the clinical significance of these changes depends on baseline TG status, which was heterogenous across included studies. We did not perform subgroup analyses by baseline TG category due to incomplete reporting in primary studies, and we recommend that future trials systematically report baseline TG stratification to enable more nuanced clinical interpretation.

#### 4.2.3 Positioning of combined exercise and resistance training

Combined exercise (CE) demonstrated moderate efficacy in this study, with a SUCRA value (60.63%) intermediate between HIIT/AE and RT. This finding appears to diverge from conclusions in existing studies reporting combined training as the most effective intervention for TG reduction (Zhang et al., 2025); however, it is essential to consider differences in population characteristics (this study was restricted to postmenopausal women, whereas the referenced study included a broader metabolic syndrome population) and outcome definitions. The metabolic advantage of CE may stem from the synergistic effects between the aerobic component-promoting fatty acid oxidation and the resistance component-induced muscle mass augmentation, which indirectly improves lipid metabolism through enhanced resting metabolic rate and insulin sensitivity (Liu et al., 2026). Nevertheless, the relatively flat dose-response curve for CE in this study suggests lower sensitivity of its efficacy to dose variations, potentially providing relatively stable benefits across a broader dose range.

RT demonstrated the most limited TG-lowering effect in this study, with effect estimates failing to exclude the null value. This finding is consistent with conclusions from some previous meta-analyses (Wewege et al., 2022), though it warrants cautious interpretation. The primary metabolic benefits of RT may manifest more prominently in body composition improvement (lean mass accretion, body fat reduction) and insulin sensitivity enhancement rather than direct modulation of circulating TG concentrations (Costa et al., 2019). Furthermore, evidence suggests that RT may upregulate lipoprotein lipase (LPL) activity, the key enzyme hydrolyzing triglyceride-rich lipoproteins (such as VLDL and chylomicrons), thereby accelerating TG clearance. A study in obese patients with type 2 diabetic nephropathy demonstrated that periodic resistance training significantly reduced blood TG levels, though this change was accompanied by concurrent improvements in body weight, glucose, and insulin resistance index (HOMA-IR) (Li et al., 2024), indicating that TG reduction resulted from optimization of the overall metabolic environment rather than an isolated RT effect. It is noteworthy that the metabolic benefits of RT may vary across populations. For instance, one study indicated that obesity may attenuate the improvement in insulin sensitivity from RT and diminish strength gains (Ahtiainen et al., 2020), suggesting that higher intensity or longer duration RT interventions may be required to achieve equivalent metabolic benefits in obese individuals.

### 4.3 Non-linear characteristics of the dose-response relationship

#### 4.3.1 Biological plausibility of the inverted u-shaped curve

A distinctive methodological contribution of this exploratory study is the application of the MBNMA framework to suggest a non-linear quadratic relationship between exercise dose and TG reduction, a pattern that, if confirmed, may possess physiological foundations. In the low-to-moderate dose range, exercise-induced lipid metabolic adaptations follow classical dose-effect incremental patterns: increased energy expenditure, enhanced fat oxidation, and reduced hepatic VLDL synthesis collectively lower circulating TG levels (Wen et al., 2024). The endocrine stress responses associated with high exercise volumes may explain the observed plateau without necessarily implying harm (Athanasiou et al., 2023); second, the chronic inflammatory state accompanying very high exercise volumes may offset some metabolic benefits of exercise (Meyer-Lindemann et al., 2023); third, postmenopausal women, due to estrogen deficiency-induced impairment in lipid metabolic flexibility, may possess limited adaptive reserves to exercise stress and be more susceptible to “overtraining”-related metabolic rebound (Sanchez et al., 2024).

#### 4.3.2 Clinical translation value of optimal dose intervals

The tentative dose intervals estimated in this study for each exercise modality if validated in future high-quality RCTs, may carry potential clinical translation value. For AE, the optimal dose of 830–1,000 MET-min/week corresponds to approximately 185–220 minutes of moderate-intensity walking (4.5 METs) or 125–150 minutes of moderate-intensity cycling (6.5 METs) per week. This exercise volume falls within the range commonly recommended for achieving general health benefits, while the present findings specifically address TG reduction in postmenopausal women (Piercy et al., 2018). The distinction between therapeutic dose and health maintenance dose aligns with the core principle of minimum effective dose in precision medicine (Wang et al., 2026), potentially enhancing exercise prescription adherence—excessive volume requirements constitute a major barrier to exercise engagement among postmenopausal women (Grazioli et al., 2023). The optimal dose interval for HIIT (1,000–1,300 MET-min/week), if calculated using a typical “4×4” protocol (4 sets × 4 minutes high-intensity intervals with 3-minute low-intensity recovery periods, 3 sessions per week), corresponds to approximately 90–120 minutes of total exercise time per week—substantially less than the time required for equivalent metabolic expenditure through traditional continuous aerobic exercise. This time-efficiency advantage holds significant practical appeal for postmenopausal women with demanding schedules. However, it should be noted that the number of HIIT-related studies included in this study was limited, and these results should be interpreted with caution.

#### 4.3.3 Comparison with previous dose-response investigations

The non-linear findings of this study resonate with recent dose-response investigations in related populations. The beneficial effects of exercise on lipid metabolism improve with increasing dose, but beyond a certain threshold, continued dose escalation fails to confer additional benefits and may even precipitate effect attenuation or adverse outcomes. This inverted U-shaped dose-response relationship exhibits remarkable consistency across diverse populations. Gonzalo-Encabo et al., based on a pooled analysis of two randomized controlled trials, clearly stated that as aerobic exercise dose increases (measured in kilocalories expended per week or MET·minutes per week), body fat percentage and triglyceride levels exhibit a decreasing trend. However, this decline is not linearly indefinite; when exercise volume reaches approximately 830–1000 MET·minutes per week, the reduction in TG plateaus, and further increases in exercise volume yield significantly diminished marginal benefits (Gonzalo-Encabo et al., 2021). The TyG index, calculated as ln, has been validated across numerous pediatric and adolescent cohorts as a reliable, cost-effective surrogate marker for insulin resistance, demonstrating strong correlations with the gold-standard hyperinsulinemic-euglycemic clamp and the Homeostatic Model Assessment of Insulin Resistance (HOMA-IR) (Brito et al., 2021). By focusing on postmenopausal women—a population at a specific physiological stage—this study not only validates the existence of this pattern but, more importantly, further refines the specific effective dose windows for different exercise modalities within this demographic.

### 4.4 Methodological innovation: application value of the MBNMA framework

The application of the MBNMA framework for dose-response analysis in this study confers significant methodological advantages over conventional “split” NMA approaches. Traditional methods treat different doses of the same intervention as discrete entities, failing to leverage structured information across doses, resulting in diminished statistical efficiency and precluding prediction of effects at untested doses (Arora et al., 2024). MBNMA addresses these limitations through parameterized dose-response functions, achieving the following breakthroughs: first, integrating direct and indirect evidence within a unified statistical framework to enhance effect estimation precision; second, characterizing the overall trend of the dose-response curve through function form selection (quadratic model in this study) rather than focusing exclusively on discrete dose points; third, enabling effect prediction at doses not tested in trials, providing a more comprehensive evidence landscape for clinical decision-making (Huang et al., 2025). The optimal selection of the quadratic model in this study (DIC: −88.9) and favorable model fit diagnostics (deviance plots, fit plots) validated the applicability of this methodological strategy in the present research context.

However, MBNMA application is not without inherent challenges. The dose standardization process (converting diverse exercise protocols to MET-min/week) relies on Compendium MET assignments and investigator judgments regarding exercise intensity, potentially introducing measurement error. Although this study employed systematic MET assignment rules and attempted to contact original authors for missing data, dose estimates for some studies inevitably retained uncertainty. Future research should explore the application of more precise individualized energy expenditure monitoring methods (such as wearable devices) in dose-response investigations.

A critical limitation of the dose standardization process is the potential for misclassification, particularly for HIIT and RT. Although MET values were assigned based on established ACSM categories and the 2024 Adult Compendium (detailed in **Supplementary Appendix 2**), the assignment of a default vigorous MET value (8.0) to HIIT cannot fully capture the heterogeneity in interval protocols (e.g., 4×4, Tabata, sprint intervals), which differ markedly in work-rest ratios and metabolic demands. Similarly, RT MET assignments based on %1RM or RPE categories may not reflect the true energy expenditure or physiological stimulus of diverse resistance protocols. Consequently, the optimal dose ranges identified in this study should be interpreted as tentative reference intervals rather than rigid clinical thresholds. Future research should explore individualized energy expenditure monitoring (e.g., wearable devices) to refine dose estimation precision.

### 4.5 Sources of heterogeneity and evidence limitations

 Substantial between-study heterogeneity was observed in this study; although addressed through random-effects modeling and sensitivity analyses, the sources of heterogeneity merit in-depth examination. Potential heterogeneity drivers include: baseline TG levels, duration since menopause, hormone replacement therapy use, concomitant dietary interventions, and exercise intervention supervision modalities. Regrettably, due to incomplete reporting of these covariates in original studies, the meta-regression in this study failed to identify statistically significant effect modifiers. This “ecological fallacy” risk underscores the inherent limitations of aggregate-data meta-analyses in exploring individual-level effect modification, and future investigations should encourage the adoption of individual participant data (IPD) meta-analytic strategies in original research.

Evidence quality limitations are equally noteworthy. CINeMA evaluation indicated that most comparisons exhibited low to very low certainty evidence, primarily constrained by the high proportion of included studies with elevated risk of bias and small-study effects. The evidence for HIIT was particularly sparse (only 3 studies); although it performed optimally in rankings, decision-makers should exercise caution regarding this conclusion based on limited evidence. Sensitivity analyses excluding high risk of bias and small studies maintained primary findings, enhancing conclusion credibility to some extent.

Menopause-specific variables: Because the study population comprised postmenopausal women, heterogeneity may derive from menopause-specific characteristics that were poorly reported in primary studies, including: type of menopause (natural vs. surgical), time since menopause, hormone replacement therapy use, vasomotor symptoms, and baseline body composition. These factors are known to modify lipid responses to exercise in postmenopausal women, yet few trials reported them comprehensively. For example, surgical menopause may confer more severe metabolic perturbations than natural menopause, potentially altering exercise responsiveness. Similarly, hormone replacement therapy can independently improve lipid profiles, potentially confounding exercise effect estimates. Future RCTs should standardize the reporting of menopause-specific variables to enable more precise effect modification analyses.

### 4.6 Clinical and public health implications

The findings of this study carry dual implications for clinical practice and public health policy in cardiovascular risk management for postmenopausal women. At the clinical level, exercise prescription should transition from “one-size-fits-all” generic recommendations toward precision schemes based on specific outcomes and individual characteristics. For postmenopausal women with TG management as the primary objective, moderate-dose AE represents the most evidence-supported option, while HIIT may be considered as a promising but experimental alternative pending further validation; for individuals with exercise contraindications or preferences for low-impact activities, CE offers an alternative with stable benefits across a broader dose range. At the public health level, the “minimum effective dose” identified in this study (clinically meaningful improvement observable at approximately 500 MET-min/week for AE) falls below WHO generic recommendations, underscoring the necessity of developing differentiated exercise guidelines targeting specific health outcomes. This may help mitigate “exercise phobia”—the behavioral pattern of abandoning exercise due to inability to meet high volume standards—which is particularly prevalent among postmenopausal women.

### 4.7 Strengths and limitations

Strengths of this study include: rigorous adherence to PRISMA-NMA guidelines; PROSPERO prospective registration; comprehensive literature search spanning 8 databases without language restrictions; systematic exercise dose standardization protocol; exploratory application of the MBNMA framework for dose-response modeling in postmenopausal women; and comprehensive sensitivity analyses and consistency assessments. Limitations include: the high proportion of included studies with elevated risk of bias; missing data on key covariates (such as dietary intake, hormone use history) constraining effect modification exploration; relatively limited number of HIIT and CE studies; restriction of all included studies to middle- and high-income countries, limiting generalizability; and inherent dose estimation error risk associated with MBNMA.

It is critical to emphasize that the dose-response curves and optimal dose intervals presented in this study should be interpreted as exploratory and hypothesis-generating rather than definitive clinical recommendations. The MBNMA framework, while methodologically innovative, relies on aggregate data from studies with predominantly low-to-moderate certainty evidence (per CINeMA assessment) and substantial risk of bias concerns (only 10 of 51 studies rated as low risk). The HIIT findings, in particular, are based on merely three studies with wide credible intervals, rendering the SUCRA ranking highly unstable. Furthermore, the dose standardization process introduces measurement error, particularly for HIIT and RT protocols. Consequently, the proposed “optimal” dose ranges should be viewed as tentative reference points for future RCT design rather than prescriptive clinical thresholds. Clinicians and policymakers should await confirmation from larger, rigorously designed trials before implementing these findings into practice.

## 5 Conclusions

This investigation provides preliminary evidence for a non-linear dose-response relationship between exercise volume and TG reduction in postmenopausal women. HIIT demonstrated the highest relative efficacy in SUCRA rankings, though this conclusion is based on limited evidence. All active interventions produced clinically meaningful TG lowering within specific dose ranges, but the observed non-linear pattern should be interpreted cautiously given the low-to-moderate certainty of evidence. These findings challenge the simplistic “more is better” paradigm and offer hypothesis-generating guidance for precision exercise prescription, pending confirmation by future high-quality RCTs.
