# Supplementary material for: Exercise dose and triglyceride reduction in postmenopausal women: a systematic review and model-based network meta-analysis
Source: Front Physiol. 2026 Sep 7;17:1911395. doi: 10.3389/fphys.2026.1911395 (PMC13591210; doi:10.3389/fphys.2026.1911395)
Supplement: Supplementary file 1 [file SupplementaryFile1.docx]

**Contents**

[Appendix 1. Search strategies	2](#_Toc29228)

[Appendix 2. Characteristics of included studies	5](#_Toc23925)

[Appendix 3. Summary of risk of bias	16](#_Toc23715)

[Appendix 4. Evidence assessment	17](#_Toc16046)

[Appendix 5. Bayesian network meta-analysis	18](#_Toc20925)

[Appendix 6. Model fit summaries for all studies and sensitivity analyses	20](#_Toc495)

[Appendix 7. Dose-response network meta-analyses	21](#_Toc10820)

[Appendix 9. References	37](#_Toc18017)

# Appendix 1. Search strategies

| Databases | Terms |
| --- | --- |
| PubMed（159） | ((("Menopause"[MeSH] OR "Estrogen Replacement Therapy"[MeSH] OR "Hot Flashes"[MeSH] OR "Osteoporosis, Postmenopausal"[MeSH] OR "Postmenopause"[MeSH] OR "Premenopause"[MeSH] OR "Perimenopause"[MeSH] OR "Climacteric"[MeSH]) OR (menopause[Title/Abstract] OR postmenopausal[Title/Abstract] OR perimenopausal[Title/Abstract] OR climacteric[Title/Abstract] OR "estrogen replacement"[Title/Abstract])) AND ((("Exercise"[MeSH] OR "Physical Exertion"[MeSH] OR "Exercise Therapy"[MeSH] OR "Resistance Training"[MeSH] OR "High-Intensity Interval Training"[MeSH] OR "Physical Fitness"[MeSH]) OR (exercise[Title/Abstract] OR "physical activity"[Title/Abstract] OR "aerobic training"[Title/Abstract] OR "aerobic exercise"[Title/Abstract] OR "endurance training"[Title/Abstract] OR "endurance exercise"[Title/Abstract] OR "resistance training"[Title/Abstract] OR "resistance exercise"[Title/Abstract] OR "strength training"[Title/Abstract] OR "strength exercise"[Title/Abstract] OR "combined training"[Title/Abstract] OR "combined exercise"[Title/Abstract] OR "concurrent training"[Title/Abstract] OR "interval training"[Title/Abstract] OR "interval exercise"[Title/Abstract] OR "sprint training"[Title/Abstract])) AND ((("Triglycerides"[MeSH] OR "Hypertriglyceridemia"[MeSH]) OR (triglyceride[Title/Abstract] OR triacylglycerol"[Title/Abstract] OR "serum triglyceride"[Title/Abstract] OR "plasma triglyceride"[Title/Abstract] OR "blood triglyceride"[Title/Abstract] OR TG[Title/Abstract])) AND (("Randomized Controlled Trial"[Publication Type] OR "randomized controlled trials as topic"[MeSH]) OR (randomized[Title/Abstract] OR randomised[Title/Abstract] OR randomly[Title/Abstract] OR trial[Title/Abstract] OR placebo[Title/Abstract])) |
| Scopus（450） | ( TITLE-ABS-KEY("Menopause" OR "Estrogen Replacement Therapy" OR "Hot Flashes" OR "Osteoporosis, Postmenopausal" OR "Postmenopause" OR "Premenopause" OR "Perimenopause" OR "Climacteric" OR menopause OR postmenopausal OR perimenopausal OR climacteric OR "estrogen replacement") ) AND ( TITLE-ABS-KEY("Exercise" OR "Physical Exertion" OR "Exercise Therapy" OR "Resistance Training" OR "High-Intensity Interval Training" OR "Physical Fitness" OR exercise OR "physical activity" OR "aerobic training" OR "aerobic exercise" OR "endurance training" OR "endurance exercise" OR "resistance training" OR "resistance exercise" OR "strength training" OR "strength exercise" OR "combined training" OR "combined exercise" OR "concurrent training" OR "interval training" OR "interval exercise" OR "sprint training") ) AND ( TITLE-ABS-KEY("Triglycerides" OR "Hypertriglyceridemia" OR triglyceride OR triacylglycerol OR "serum triglyceride" OR "plasma triglyceride" OR "blood triglyceride" OR TG) ) AND ( TITLE-ABS-KEY("Randomized Controlled Trial" OR "randomized controlled trials as topic" OR randomized OR randomised OR randomly OR trial OR placebo) ) |
| The Cochrane Library（205） | ((Menopause OR "Estrogen Replacement Therapy" OR "Hot Flashes" OR "Osteoporosis, Postmenopausal" OR Postmenopause OR Perimenopause):[MeSH] OR (menopause OR postmenopausal OR perimenopausal OR climacteric):ti,ab,kw) AND ((Exercise OR "Physical Exertion" OR "Exercise Therapy" OR "Resistance Training" OR "High-Intensity Interval Training" OR "Physical Fitness"):[MeSH] OR (exercise OR "physical activity" OR "aerobic training" OR "aerobic exercise" OR "endurance training" OR "endurance exercise" OR "resistance training" OR "resistance exercise" OR "strength training" OR "strength exercise" OR "combined training" OR "concurrent training" OR "interval training" OR "sprint training"):ti,ab,kw) AND ((Triglycerides OR Hypertriglyceridemia):[MeSH] OR (triglyceride OR triacylglycerol OR "serum triglyceride" OR "plasma triglyceride"):ti,ab,kw) |
| Web of Science  （173） | TS=("Menopause" OR "Estrogen Replacement Therapy" OR "Hot Flashe" OR "Osteoporosis, Postmenopausal" OR "Postmenopause" OR "Premenopause" OR "postmenopausal" OR "perimenopause" OR "climacteric") AND TS=("Exercise" OR "Physical Exertion" OR "Exercise Therapy" OR "Resistance Training" OR "High-Intensity Interval Training" OR "physical activity" OR "aerobic training" OR "aerobic exercise" OR "endurance training" OR "endurance exercise" OR "resistance exercise" OR "strength training" OR "strength exercise" OR "combined training" OR "combined exercise" OR "concurrent training" OR "interval training" OR "interval exercise" OR "sprint training") AND TS=("Triglyceride" OR "Triacylglycerol" OR "Triacylglycerols" OR "triglycerides" OR "TG" OR "serum triglyceride" OR "plasma triglyceride" OR "blood triglyceride") AND TS=("randomized controlled trial" OR "randomised controlled trial" OR "randomized" OR "randomised" OR "randomly" OR "placebo" OR "RCT") |
| China National Knowledge Infrastructure（51） | (SU='menopause' OR SU='climacteric' OR SU='perimenopause' OR SU='menopause' OR SU='postmenopause' OR SU='estrogen replacement therapy' OR SU='hot flashes' OR SU='osteoporosis' OR TKA='menopause' OR TKA='climacteric' OR TKA='perimenopause' OR TKA='menopause' OR TKA='postmenopause') AND (SU='exercise' OR SU='physical exercise' OR SU='physical activity' OR SU='physical exercise' OR SU='aerobic exercise' OR SU='endurance exercise' OR SU='resistance training' OR SU='strength training' OR SU='high-intensity interval training' OR SU='physical activity' OR TKA='exercise' OR TKA='physical exercise' OR TKA='physical activity' OR TKA='physical exercise' OR TKA='aerobic exercise' OR TKA='endurance exercise' OR TKA='resistance training' OR TKA='strength training' OR TKA='high-intensity interval training' OR TKA='physical activity' OR TKA='physical activity') AND (SU='triglycerides' OR SU='triacylglycerol' OR SU='lipids' OR SU='hypertriglyceridemia' OR TKA='triglycerides' OR TKA='triacylglycerol' OR TKA='lipids' OR TKA='TG') AND (SU='randomized controlled trial' OR SU='RCT' OR SU='clinical trial' OR TKA='randomized' OR TKA='controlled' OR TKA='RCT' OR TKA='randomized controlled') |
| Wanfang Database  （70） | (Subject: ("menopause" OR "climacteric" OR "perimenopause" OR "menopausal period" OR "postmenopause" OR "estrogen replacement therapy" OR "hot flash" OR "osteoporosis" OR "perimenopausal syndrome"))AND (Subject: ("exercise" OR "physical activity" OR "sports" OR "physical training" OR "aerobic exercise" OR "endurance training" OR "resistance training" OR "strength training" OR "high-intensity interval training" OR "physical activity" OR "body movement" OR "fitness")))AND (Subject: ("triglyceride" OR "triacylglycerol" OR "blood lipid" OR "hypertriglyceridemia" OR "TG"))AND (Subject: ("randomized controlled trial" OR "RCT" OR "randomized" OR "controlled" OR "clinical trial")) |
| Weipu  Database  (27） | (M=menopause OR M=climacteric OR M=perimenopause OR M=menopausal period OR M=postmenopause OR M=estrogen replacement therapy OR M=hot flash OR M=osteoporosis OR M=perimenopausal syndrome OR U=menopause OR U=climacteric OR U=perimenopause) AND (M=exercise OR M=physical training OR M=physical activity OR M=physical exercise OR M=aerobic exercise OR M=endurance training OR M=resistance training OR M=strength training OR M=high-intensity interval training OR M=physical activity OR M=body movement OR M=fitness OR U=exercise OR U=physical training OR U=physical activity) AND (M=triglyceride OR M=triacylglycerol OR M=blood lipid OR M=hypertriglyceridemia OR M=TG OR U=triglyceride OR U=triacylglycerol OR U=blood lipid) AND (M=randomized controlled trial OR M=RCT OR M=randomized OR M=controlled OR M=clinical trial OR U=randomized OR U=controlled OR U=RCT) |
| Chinese Biomedical Database  (39) | ("menopause"[Common Field: Smart] OR "climacteric"[Common Field: Smart] OR "perimenopause"[Common Field: Smart] OR "menopausal period"[Common Field: Smart] OR "postmenopause"[Common Field: Smart] OR "estrogen replacement therapy"[Common Field: Smart] OR "hot flash"[Common Field: Smart] OR "osteoporosis, postmenopausal"[Common Field: Smart]) AND ("exercise"[Common Field: Smart] OR "physical training"[Common Field: Smart] OR "physical activity"[Common Field: Smart] OR "physical exercise"[Common Field: Smart] OR "exercise therapy"[Common Field: Smart] OR "aerobic exercise"[Common Field: Smart] OR "endurance training"[Common Field: Smart] OR "resistance training"[Common Field: Smart] OR "strength training"[Common Field: Smart] OR "high-intensity interval training"[Common Field: Smart] OR "physical activity"[Common Field: Smart]) AND ("triglyceride"[Common Field: Smart] OR "triacylglycerol"[Common Field: Smart] OR "blood lipid"[Common Field: Smart] OR "hypertriglyceridemia"[Common Field: Smart] OR "TG"[Common Field: Smart]) AND ("randomized controlled trial"[Publication Type] OR "randomized controlled trial"[Common Field: Smart] OR "RCT"[Common Field: Smart] OR "randomized"[Common Field: Smart] OR "controlled"[Common Field: Smart]) |

# Appendix 2. Characteristics of included studies

**Table S1.** Characteristics of included studies

| **Author/Year** | **Characteristics** | **Intervention** | **Age** | **BMI** | **Intervention** | **Briefdescription** | **Met**  **(min/week)** |
| --- | --- | --- | --- | --- | --- | --- | --- |
|  |  | **Control** |  |  |  |  |  |
| Ağl et al. 2010 | Healthy, postmenopausal women | 18 | 52.4 ± 4.7 | NR | AE | Cycle ergometer, intensity determined by submaximal exercise test. | 1036 |
|  |  | 18 | 52.6 ± 3.5 | NR | RT | Elastic band exercises (major muscle groups), 10-15 reps per set. | 356 |
| Akazawa et al. 2012 | Healthy, sedentary, postmenopausal women | 10 | 60 ± 2 | NR | AE | Walking or cycling, intensity gradually increasing from 60% of maximal heart rate to 70-75%. | 641 |
|  |  | 10 | 61 ± 2 | NR | CON |  | 0 |
| Akazawa et al. 2012 | Healthy, sedentary, postmenopausal women | 11 | 59 ± 5 | 22.7 ± 1.0 | AE | Walking or cycling, intensity gradually increasing from 60% of maximal heart rate to 70-75%. | 800 |
|  |  | 10 | 64 ± 6 | 21.5 ± 1.0 | CON |  | 0 |
| Akazawa et al. 2013 | Healthy, sedentary, postmenopausal women | 13 | 60 ± 1 | 21.5 ± 0.8 | AE | Walking or cycling, intensity gradually increasing from 60% of maximal heart rate to 70-75%. | 1935 |
|  |  | 12 | 58 ± 1 | 22.1 ± 0.5 | CON |  | 367 |
| Arca et al. 2014 | Hypertensive, postmenopausal women | 19 | 64 ± 7 | 27.0 ± 5.1 | AE | 10 minutes stretching + 20 minutes main training. | 0 |
|  |  | 19 | NR | 28.3 ± 4.2 | AE | 10 minutes stretching + 40 minutes main training. | 1385 |
|  |  | 14 | NR | 30.9 ± 4.8 | CON |  | 0 |
| Binder et al. 1996 | Healthy, sedentary, postmenopausal women | 23 | 65 ± 4 | NR | AE | First 2 months flexibility training, then 9 months endurance training (walking, jogging, stair climbing), intensity 60-85% VO_2_Max. | 447 |
|  |  | 17 | 67 ± 4 | NR | CON |  | 0 |
| Buttelli et al. 2020 | Postmenopausal women with dyslipidemia | 20 | 64.20 ± 2.53 | 30.54 ± 3.53 | AE | Mat Pilates, including basic exercises and fundamental Pilates movements, 2-4 times per week, 45-55 minutes per session, for 10 weeks. | 628 |
|  |  | 6 | 64.16 ± 3.05 | 33.38 ± 3.78 | CON |  | 0 |
| Chagas et al. 2017 | Obese, postmenopausal women | 35 | 61.3 ± 6.4 | 30.6 ± 3.5 | AE | Level walking at 50-60% VO₂peak. | 950 |
|  |  | 35 | 59.8 ± 7.1 | 32.8 ± 3.4 | CON |  | 1250 |
| Colado et al. 2009 | Healthy, sedentary, postmenopausal women | 15 | 54.7 ± 2.0 | 27.6 ± 3.7 | RT | Use of water-based resistance devices (gloves, paddles, bells, etc.), same periodized training as EB group. | 0 |
|  |  | 21 | 54.0 ± 2.8 | 29.5 ± 3.3 | RT | Use of elastic bands, same periodized training. Both groups perform 8-10 total-body exercises. | 1805 |
|  |  | 10 | 52.9 ± 1.9 | 27.5 ± 3.3 | CON |  | 0 |
| Dupuit et al. 2022 | Overweight/obese, postmenopausal women | 8 | 58.8 ± 5.3 | 30.3 ± 3.5 | CE | Aerobic (60% heart rate reserve) and resistance training. | 748 |
|  |  | 9 | 60.9 ± 4.8 | 31.5 ± 3.4 | CON |  | 0 |
| Diniz et al. 2015 | Postmenopausal women | 10 | 60.26 ± 8.51 | 25.82 ± 2.74 | AE | Running/brisk walking at 100% critical velocity, 50 minutes per session. | 550 |
|  |  | 9 | 62.01 ± 7.83 | 25.76 ± 2.93 | CON |  | 0 |
| Elliott et al. 2002 | Sedentary, postmenopausal women | 8 | 58 ± 4 | 26.89 ± 5.94 | RT | 5 resistance exercises (leg press, bench press, leg extension, leg curl, seated pulldown), intensity 80% of 10RM, 8 reps per set. | 1177 |
|  |  | 7 | 53 ± 3 | 23.97 ± 2.58 | CON |  | 0 |
| Frank et al. 2005 | Sedentary, overweight/obese, postmenopausal women | 87 | 60.7 ± 6.7 | 30.4 ± 4.1 | AE | Treadmill walking and outdoor walking/cycling, intensity gradually increasing from 40% of maximal heart rate to 60-75%, 5 times per week, 45 minutes per session. | 625 |
|  |  | 86 | 60.6 ± 6.8 | 30.5 ± 3.7 | CON |  | 0 |
| Gómez-Tomás et al. 2018 | Sedentary, postmenopausal women | 18 | 70.89 ± 4.42 | 28.72 ± 4.48 | RT | 6 total-body resistance exercises (using elastic bands), divided into 3 phases (every 4 months), intensity increased according to OMNI-RES scale (levels 3-7) and band color (yellow, red, green), 10 reps per set. | 851 |
|  |  | 20 | 70.45 ± 5.44 | 30.16 ± 5.57 | CON |  | 0 |
| Ha et al. 2021 | Elderly women with metabolic syndrome | 6 | 75.8 ± 4.3 | 23.94 ± 1.54 | AE | Water-based activity, intensity 30-60% of heart rate reserve. | 1896 |
|  |  | 7 | 76.7 ± 5.9 | 24.78 ± 1.06 | CON |  | 1206 |
| He et al. 2022 | Healthy, sedentary, postmenopausal women | 10 | 55.8 ± 5.6 | 23.2 ± 0.6 | HIIT | Running, 4 sets of 4 minutes high intensity (85-95% of maximal heart rate) interspersed with 3 minutes low intensity (60-70% of maximal heart rate). | 0 |
|  |  | 15 | 57.6 ± 3.25 | 23.8 ± 0.4 | AE | Running at 40% of heart rate reserve. | 845 |
|  |  | 15 | 54.3 ± 6.4 | 24.9 ± 0.7 | AE | Running at 70-80% of maximal heart rate. | 0 |
| Heli V et al. 2013 | Postmenopausal women with metabolic syndrome | 6 | 63.3 ± 9.6 | 30.8 ± 3.9 | CE | Interval aerobic (cycle ergometer, intensity 50-80% of heart rate reserve) and resistance training (bodyweight and dumbbells). | 752 |
|  |  | 4 | 55.8 ± 6.1 | 34.9 ± 6.2 | CON |  | 0 |
| Keawtep et al. 2024 | Postmenopausal women with obesity | 23 | 52.70±3.60 | 29.06 ± 2.90 | CE | Home-based physical-cognitive training (aerobic + resistance + cognitive tasks), 3 times per week, 60 minutes per session. | 1732 |
|  |  | 23 | 53.61±2.81 | 29.18 ± 2.85 | CON |  | 0 |
| Kemmler et al. 2009 | Elderly women with metabolic syndrome | 33 | 68.7 ± 3.4 | 27.5 ± 3.4 | HIIT | High-intensity multicomponent training, including aerobic dance, coordination training, isometric and dynamic strength exercises. | 842 |
|  |  | 32 | 69.5 ± 4.3 | 28.9 ± 4.5 | CON |  | 0 |
| Kim & Kim 2012 | Obese, postmenopausal women | 15 | 54.53 ± 2.82 | 25.03 ± 1.33 | AE | Line dance, intensity gradually increasing from 55-65% of maximal heart rate to 70-80%, 3 times per week, 60 minutes per session. | 787 |
|  |  | 15 | NR | 25.12 ± 1.49 | CON |  | 0 |
| Kheirat et al. 2017 | Postmenopausal women adherent to Mediterranean diet | 30 | 55 ± 4 | 23.80 ± 1.05 | AE | Aerobic exercise at 60% of maximal heart rate, 3 times per week, 60 minutes per session. | 990 |
|  |  | 35 | 56 ± 2 | 24.13 ± 0.78 | CON |  | 457 |
| Lee et al. 2021 | Obese, postmenopausal women | 12 | 56.0 ± 2.9 | 25.8 ± 2.0 | AE | Taekwondo training, including poomsae, kicking, footwork drills, and Taekwondo aerobics, intensity 50-80% of maximal heart rate. | 0 |
|  |  | 12 | 57.5 ± 2.9 | 25.5 ± 1.7 | CON |  | 1867 |
| Lee et al. 2017 | Obese, postmenopausal women | 18 | 55.00 ± 1.86 | 25.60 ± 1.63 | AE | Line dance, intensity gradually increasing from 50-60% of maximal heart rate to 70-80%. | 0 |
|  |  | 18 | 54.10 ± 1.95 | 26.94 ± 2.77 | CON |  | 1135 |
| Lee et al. 2012 | Obese, postmenopausal women | 8 | 54.75 ± 2.76 | 25.13 ± 1.63 | AE | Yoga practice, including postures, breathing techniques, and relaxation. | 0 |
|  |  | 8 | 54.25 ± 2.91 | 25.19 ± 1.71 | CON |  | 462 |
| Maesta et al. 2007 | Postmenopausal women | 11 | 60.7 ± 7.1 | 27.7 ± 4.4 | RT | 8 resistance exercises (leg press, leg extension, bench press, seated row, etc.), intensity 60-80% 1RM, 8-12 reps per set. | 0 |
|  |  | 11 | 57.9 ± 6.9 | 26.6 ± 3.6 | CON |  | 797 |
| Mohanka et al. 2006 | Sedentary, overweight/obese, postmenopausal women | 87 | 60.6 ± 6.6 | NR | AE | Treadmill walking and stationary cycling, intensity 60-75% of maximal heart rate, 5 times per week, 45 minutes per session. | 0 |
|  |  | 86 | 60.5 ± 6.7 | NR | CON |  | 924 |
| Miyaki et al. 2012 | Healthy, sedentary or recreationally active, postmenopausal women | 11 | 60 ± 6 | 22.2 ± 2.0 | AE | Walking or cycling, intensity 60-75% of maximal heart rate, 3-5 times per week, 30-45 minutes per session. | 0 |
|  |  | 11 | 60 ± 7 | 22.4 ± 2.6 | CON |  | 715 |
| Najafi Nazarabadi et al. 2022 | Postmenopausal women with metabolic syndrome | 12 | 45.25 ± 2.22 | 33.94 ± 1.24 | AE | Treadmill walking/jogging, intensity 60-75% of maximal heart rate, 20-40 minutes main training per session. | 0 |
|  |  | 11 | 56.36 ± 1.91 | 34.41 ± 1.01 | CON |  | 420 |
| Nishida et al. 2015 | Healthy, postmenopausal women | 31 | 70.4 ± 5.8 | 24.2 ±3.7 | AE | Home-based step exercise at lactate threshold level, 10-20 minutes per session, 3 times per day, target ≥140 minutes per week. | 0 |
|  |  | 31 | 69.7 ± 6.6 | 22.2 ± 2.5 | CON |  | 1820 |
| Paolillo et al. 2017 | sedentary, postmenopausal women | 10 | 55 ± 2 | NR | AE | Treadmill training at 85-90% of maximal heart rate, 45 minutes per session. | 0 |
|  |  | 10 | 55 ± 2 | NR | CON |  | 891 |
| Park et al. 2015 | Postmenopausal women with abdominal obesity | 10 | 57.20 ± 2.57 | 26.02 ± 1.55 | CE | Resistance part: 10 exercises (arm curls, leg extensions, squats, bench press, etc.), intensity 60-70% 1RM, 8-12 reps per set, 3 sets, 30 minutes." | 0 |
|  |  | 10 | 57.20 ± 1.69 | 26.80 ± 1.09 | CON |  | 1800 |
| Pereira et al. 2023 | Inactive, postmenopausal women | 31 | 64 ± 7 | NR | CE | Includes warm-up (low-intensity running, coordination training, strength, flexibility, balance exercises), followed by 3×15 minutes of small-sided handball matches (4v4, 5v5, 6v6), intensity monitored by heart rate. | 0 |
|  |  | 14 | 67 ± 5 | NR | CON |  | 382 |
| Pospieszna et al. 2017 | Healthy, postmenopausal women | 20 | 62 ± 3.8 | 25.06 ± 2.49 | AE | Nordic walking, intensity at 90% of ventilatory threshold, heart rate monitoring for intensity control. | 0 |
|  |  | 19 | 62 ± 1.1 | 26.04 ± 0.83 | CON |  | 924 |
| Rossi et al. 2016 | Postmenopausal women | 13 | 61.8 ± 7.9 | NR | AE | Running or brisk walking at 100% of critical velocity. | 798 |
|  |  | 16 | 61.4 ± 5.0 | NR | CE | Resistance training (9 exercises, 3 sets, 8-15 reps per set, intensity to failure) + aerobic training (30 minutes at 100% critical velocity). | 560 |
|  |  | 10 | 63.4 ± 7.5 | NR | CON |  | 330 |
| Ready et al. 1996 | Sedentary, postmenopausal women | 19 | 61.3 ± 5.8 | NR | AE | Walking at 60% of VO_2_peak, 60 minutes per session. Approximately 300 minutes per week. | 0 |
|  |  | 17 | NR | NR | CON |  | 506 |
| Seo et al. 2014 | Healthy, sedentary, postmenopausal women | 8 | 54.4 ± 5.4 | 24.0 ± 2.4 | CE | Combined aerobic + resistance training at 60% of predicted maximal heart rate. | 0 |
|  |  | 6 | 54.4 ± 5.4 | 24.0 ± 2.4 | CON |  | 680 |
| Rossi et al. 2017 | Postmenopausal women | 20 | 62.2 ± 6.3 | NR | CE | Resistance training (9 exercises, 3 sets, 12-15 reps or 10-12 reps) + aerobic training (100% critical velocity). | 0 |
|  |  | 15 | 61.9 ± 7.2 | NR | CON |  | 525 |
| Rossi et al. 2017 | Postmenopausal women | 8 | 60.6 ± 7.9 | NR | AE | Running/brisk walking at 100% critical velocity. | 0 |
|  |  | 15 | 62.4 ± 5.1 | NR | CE | Resistance training (9 exercises, 3 sets, 8-15 reps) + aerobic training (100% critical velocity). | 622 |
|  |  | 8 | 62.8 ± 5.9 | NR | CON |  | 0 |
| Soori et al. 2017 | Sedentary, obese, postmenopausal women | 8 | 45-60 | BMI ≥ 30 kg/m² | AE | Swimming or walking in water, starting at 40% of maximal heart rate, increasing by 5% every two weeks. | 800 |
|  |  | 8 |  |  | RT | Free weights, 6 exercises (e.g., bench press, rowing, leg press, etc.), intensity 40% 1RM, increasing to 60% 1RM every four weeks, 10-12 reps per set, 3 sets total. | 0 |
|  |  | 8 |  |  | CE | Resistance training (2 sets, 40% 1RM) + endurance training (40% of maximal heart rate). | 586 |
|  |  | 8 |  |  | CON |  | 718 |
| Shamlou Kazemi et al. 2023 | Postmenopausal women with metabolic syndrome | 15 | 45-65 | 28.33 ± 0.36 | HIIT | 3 minutes high intensity (80-90% of maximal heart rate) + 3 minutes active recovery (55-65% of maximal heart rate) | 0 |
|  |  | 15 |  | 27.94 ± 0.71 | RT | 10 exercises (leg press, bench press, leg extension, etc.), intensity 75-80% 1RM, 8-10 reps per set, 3 sets. | 1110 |
|  |  | 15 |  | 27.14 ± 0.38 | CON |  | 0 |
| Wang et al. 2018 | Older women with type 2 diabetes | 16 | 60-69 | NR | AE | Walking or jogging at individualized FATmax intensity (maximal fat oxidation intensity, average 37% VO_2_Maax). | 1056 |
|  |  | 15 |  | NR | CON |  | 0 |
| Wen et al. 2016 | Postmenopausal women with low bone mass | 24 | 57.5 ± 3.5 | 21.69 ± 2.94 | AE | Group step aerobics under professional instructor guidance, intensity 75-85% of heart rate reserve, step height gradually increasing from 10 cm to 20-25 cm. | 805 |
|  |  | 22 | 58.8 ± 3.2 | 22.27 ± 1.89 | CON |  | 0 |
| Wooten et al. 2011 | Sedentary, obese, postmenopausal women | 9 | 64.4 ± 0.7 | 31.0 ± 0.5 | RT | 10 total-body resistance exercises (e.g., bench press, leg press, leg extension, etc.), intensity 8-RM, 2 sets of 8 reps per exercise and 1 set to failure. | 377 |
|  |  | 12 | 67.0 ± 0.6 | 34.0 ± 1.3 | CON |  | 858 |
| Wu et al. 2006 | Healthy, early postmenopausal women | 31 | 55.2 ± 2.8 | 22.4 ± 2.9 | AE | Supervised walking 3 times per week, speed 5-6 km/h. | 0 |
|  |  | 33 | 54.9 ± 2.9 | 20.9 ± 2.2 | CON |  | 719 |
| Yoshizawa et al. 2010 | Healthy, sedentary, postmenopausal women | 33 | 57 ± 1 | 23.7 ± 0.9 | AE | Moderate-intensity aerobic exercise (walking or cycling), intensity gradually increasing from 60% of maximal heart rate to 70-75%, frequency from 3-4 days per week to 4-5 days per week, duration from 25-30 minutes to 40-45 minutes per session. | 0 |
|  |  | 10 | 56 ± 1 | 23.0 ± 0.8 | CON |  | 857 |
| Yoon et al. 2018 | Obese, postmenopausal women | 10 | 53.70 ± 3.37 | 24.16 ± 2.21 | AE | Treadmill walking, intensity 60%-80% of heart rate reserve. | 839 |
|  |  | 10 | 52.20 ± 2.15 | 24.31 ± 1.65 | RT | 9 exercises (e.g., bench press, seated row, leg press, etc.), intensity 60% 1RM, 8-12 reps per set, 3 sets. | 730 |
|  |  | 10 | 52.50 ± 2.68 | 23.94 ± 2.18 | CON |  | 0 |
| Huang Caihua et al. 2008 | Obese, postmenopausal women | 16 | 52.5 ± 3.1 | 27.51 ± 1.72 | AE | Brisk walking, jogging, aerobic dance, intensity 60-70% of maximal heart rate. | 873 |
|  |  | 12 | NR | 27.88 ± 2.34 | RT | Same aerobic exercise + 20 minutes resistance training (bodyweight and light equipment, 4 exercises, 25 reps per set). | 1005 |
|  |  | 11 | NR | 26.96 ± 2.54 | CON |  | 0 |
| Chen Chunyang et al. 2013 | Obese, postmenopausal women | 16 | 57.3 | 26. 98±2. 12 | AE | Brisk walking, jogging, aerobic dance, intensity gradually increasing from 50-60% of maximal heart rate to 65-75%. | 728 |
|  |  | 18 | 57.3 | 28. 11±2. 08 | RT | Same aerobic exercise + resistance training (bodyweight and light equipment, 3 sets, 20 reps per set). | 1187 |
|  |  | 13 | 57.3 | 24. 11±1. 84 | CON |  | 0 |
| Wang Guiran et al. 2016 | Postmenopausal breast cancer survivors | 14 | 50-65 | 21.2 ± 7.4 | CE | Intensity gradually increasing from 45% target heart rate to 65%; resistance training: 9 exercises (squat, leg press, bench press, etc.), 3 sets, 10-14 repetition maximum per set. | 1171 |
|  |  | 15 | 50-65 | 27.4 ± 3.4 | CON |  | 0 |
| Huang Caihua et al. 2009 | Postmenopausal women with type 2 diabetes mellitus | 11 | 54.09 ± 4.32 | 26.70 ± 2.84 | CE | "Aerobic part: brisk walking, jogging, aerobic dance, intensity 60-70% of maximal heart rate.Resistance part: bodyweight and light equipment, 4 exercises, 25 reps per set, 2-3 sets." | 700 |
|  |  | 10 | 53.20 ± 2.66 | 27.07 ± 2.40 | CON |  | 0 |
| Deng Xianqiang et al. 2006 | Postmenopausal women (1-10 years) | 23 | 58.4 ± 5.1 | 16.2-28.9 | AE | Brisk walking, accumulating at least 30 minutes of formal exercise daily. | 941 |
|  |  | 17 | NR | NR | CON |  | 0 |

# Appendix 3. Summary of risk of bias

**Figure S1.** Summary of risk of bias

# Appendix 4. Evidence assessment

**Figure S2.** Evidence assessment

# Appendix 5. Bayesian network meta-analysis

## 5.1 Model fit summaries for included studies

**Table S2. Model fit summaries for included studies**

| **Model** | **TG (99 data points)** | | | |
| --- | --- | --- | --- | --- |
|  | **DIC** | **pD** | **Residual**  **Deviance** | **SD (95%CrI)** |
| FE Model | 308.8 | 50.0 | 258.8 | - |
| RE Model | 157.1 | 69.2 | 87.9 | 0.14 (0.09, 0.20) |
| RE UME | 160.1 | 71.0 | 89.1 | - |

## 5.2 Node-split for all studies

**Table S3.** Node-split for all studies

| **Comparison** | **p.value** | **95%CrI** |
| --- | --- | --- |
| d.AE.CE | 0.535 |  |
| -> direct |  | -0.051 (-0.31, 0.22) |
| -> indirect |  | 0.054 (-0.14, 0.23) |
| -> network |  | 0.028 (-0.12, 0.17) |
| d.AE.CON | 0.4532 |  |
| -> direct |  | 0.19 (0.11, 0.27) |
| -> indirect |  | 0.33 (-0.025, 0.69) |
| -> network |  | 0.20 (0.13, 0.27) |
| d.AE.HIIT | 0.6646 |  |
| -> direct |  | -0.080 (-0.40, 0.23) |
| -> indirect |  | 0.011 (-0.27, 0.29) |
| -> network |  | -0.031 (-0.24, 0.17) |
| d.AE.RT | 0.192 |  |
| -> direct |  | 0.28 (-0.017, 0.56) |
| -> indirect |  | 0.042 (-0.15, 0.24) |
| -> network |  | 0.099 (-0.057, 0.26) |
| d.CE.RT | 0.296 |  |
| -> direct |  | 0.30 (-0.17, 0.78) |
| -> indirect |  | 0.020 (-0.19, 0.24) |
| -> network |  | 0.072 (-0.12, 0.27) |
| d.CON.HIIT | 0.9812 |  |
| -> direct |  | -0.28 (-0.71, 0.15) |
| -> indirect |  | -0.28 (-0.60, 0.034) |
| -> network |  | -0.23 (-0.44, -0.028) |
| d.CON.RT | 0.0544 |  |
| -> direct |  | -0.14 (-0.32, 0.033) |
| -> indirect |  | 0.37 (-0.12, 0.87) |
| -> network |  | -0.10 (-0.25, 0.051) |
| d.HIIT.RT | 0.4784 |  |
| -> direct |  | 0.050 (-0.26, 0.37) |
| -> indirect |  | 0.21 (-0.094, 0.52) |
| -> network |  | 0.13 (-0.081, 0.35) |

## 5.3 SUCRA table for all studies

**Table S4.** SUCRA table for all studies

| **Treatment** | **[,1]** | **[,2]** | **[,3]** | **[,4]** | **[,5]** | **SUCRA** |
| --- | --- | --- | --- | --- | --- | --- |
| AE | 0.2449 | 0.4727 | 0.2411 | 0.0413 | 0.0000 | 73.03% |
| CE | 0.1834 | 0.2625 | 0.3538 | 0.1963 | 0.0040 | 60.63% |
| CON | 0.0000 | 0.0001 | 0.0043 | 0.1010 | 0.8946 | 2.75% |
| HIIT | 0.5461 | 0.1839 | 0.1832 | 0.0751 | 0.0117 | 79.44% |
| RT | 0.0256 | 0.0808 | 0.2176 | 0.5863 | 0.0897 | 34.16% |

# Appendix 6. Model fit summaries for all studies and sensitivity analyses

**Table S5.** Model fit summaries for all studies and sensitivity analyses

| **Model** | **TG (99 data points)** | | | |
| --- | --- | --- | --- | --- |
|  | **DIC** | **pD** | **Residual**  **Deviance** | **SD(95%CrI)** |
|  | **All studies (99 data points)** | | | |
| FE Model | 308.8 | 50.0 | 258.8 | - |
| RE Model | 157.1 | 69.2 | 87.9 | 0.14 (0.09, 0.20) |
| RE UME | 160.1 | 71.0 | 89.1 | - |
|  | **Exclude high risk of bias (74 data points)** | | | |
| FE Model | 117.7 | 38 | 79.8 | - |
| RE Model | 116.8 | 45 | 71.8 | 0.07 (0.01, 0.15) |
| RE UME | 120 | 47.4 | 72.6 | - |
|  | **85 data points** | | | |
| FE Model | 117.7 | 38 | 79.8 | - |
| RE Model | 116.8 | 45 | 71.8 | 0.07 (0.01, 0.15) |
| RE UME | 120 | 47.4 | 72.6 | - |

***Note:*** CrI, credible interval. DIC, deviance information criterion. FE, fixed effects. pD, number of effective parameters. SD, standard deviation. RE, random effects. UME, unrelated mean effects.

# Appendix 7.Dose-response network meta-analyses

## 7.1 Key assumptions for Network Meta-Analysis

### 7.1.1 Network Connectivity

Connectivity is a key assumption in NMA that, if considered insufficient (i.e., due to lack of direct comparisons), may lead to low statistical power and misleading results ^2^. Our study assessed network connectivity at the motor and dose levels and did not find any evidence of network unconnectedness (Figure S9 and Figure S10)

**Figure S3.** Treatment-level network. The first value indicates the specific intervention and the second one is the corresponding dose of that intervention.

**Figure S4.** Agent-level network. The first value indicates the specific intervention and the second one is the corresponding dose of that intervention.

### 7.1.2 Data consistency

We performed a consistency analysis of the data by comparing the consistency of the network (i.e., network effect size) with the unrelated mean effects (UME) model (i.e., pairwise effect sizes). In practice, we checked whether the bias, the number of estimated parameters in the network, and the Deviance Information Criterion (DIC) metric were similar for both models, indicating a good fit. The comparison of these parameters showed a good agreement between the different models **(Table S9)**.

**Table S6.** Consistent and UME models fit comparison.

| **Model** | **pD** | **Deviance** | **Residual deviance** | **DIC** | **SD** |
| --- | --- | --- | --- | --- | --- |
| Consistent | 69.6 | -160.450 | 87.949 | -91.2 | 0.140 |
| UME | 69.7 | -163.301 | 85.098 | -93.2 | 0.135 |

### 7.1.3 Network transitivity

NMAs are based on the assumption of indirect/mixed comparisons, which implies that estimates of treatment effects from direct and indirect evidence are consistent but with the usual variation of meta-analyses under a random effects model ^4^. This assumption is equivalent to heterogeneity in a "standard" meta-analysis ^5^. Following a previous proposal ^6^, anomalies were assessed at a deeper network level (i.e., at the treatment level). We assessed the span by the MBNMA node-splitting method. This method splits the contribution of a specific treatment contrast into direct and indirect evidence and compares them ^7^. Similar effects indicate good span. **Table S10 and Figure S11** presents the results for transitivity in this meta-analysis.

**Table S7.** Node-splitting analysis of consistency

| **Comparison** | **p-value** | **Median** | **2.50%** | **97.50%** |
| --- | --- | --- | --- | --- |
| RT_750 vs AE_750 | 0.069 |  |  |  |
| -> direct |  | 0.554 | 0.062 | 1.066 |
| -> indirect |  | -0.085 | -0.295 | 0.126 |
| -> MBNMA |  | 0.025 | -0.181 | 0.228 |
| HIIT_1000 vs AE_750 | 0.757 |  |  |  |
| -> direct |  | -0.084 | -0.391 | 0.228 |
| -> indirect |  | -0.01 | -0.251 | 0.227 |
| -> MBNMA |  | -0.035 | -0.217 | 0.15 |
| RT_1250 vs Placebo_0 | 0.477 |  |  |  |
| -> direct |  | -0.263 | -1.443 | 0.929 |
| -> indirect |  | -0.202 | -0.568 | 0.139 |
| -> MBNMA |  | -0.209 | -0.554 | 0.126 |
| RT_1000 vs Placebo_0 | 0.358 |  |  |  |
| -> direct |  | -0.482 | -1.04 | 0.085 |
| -> indirect |  | -0.088 | -0.398 | 0.213 |
| -> MBNMA |  | -0.167 | -0.443 | 0.101 |
| RT_500 vs Placebo_0 | 0.567 |  |  |  |
| -> direct |  | -0.138 | -0.328 | 0.06 |
| -> indirect |  | -0.024 | -0.206 | 0.156 |
| -> MBNMA |  | -0.084 | -0.221 | 0.05 |
| RT_250 vs Placebo_0 | 0.342 |  |  |  |
| -> direct |  | -0.01 | -0.377 | 0.355 |
| -> indirect |  | -0.042 | -0.11 | 0.029 |
| -> MBNMA |  | -0.042 | -0.111 | 0.025 |
| HIIT_1500 vs Placebo_0 | 0.869 |  |  |  |
| -> direct |  | -0.278 | -0.729 | 0.161 |
| -> indirect |  | -0.28 | -0.612 | 0.093 |
| -> MBNMA |  | -0.279 | -0.55 | 0.012 |
| CE_1250 vs Placebo_0 | 0.494 |  |  |  |
| -> direct |  | -0.174 | -0.411 | 0.066 |
| -> indirect |  | -0.355 | -0.67 | -0.063 |
| -> MBNMA |  | -0.255 | -0.447 | -0.067 |
| CE_750 vs Placebo_0 | 0.353 |  |  |  |
| -> direct |  | -0.261 | -0.484 | -0.049 |
| -> indirect |  | -0.102 | -0.237 | 0.027 |
| -> MBNMA |  | -0.153 | -0.268 | -0.04 |
| CE_500 vs Placebo_0 | 0.393 |  |  |  |
| -> direct |  | -0.011 | -0.274 | 0.246 |
| -> indirect |  | -0.111 | -0.19 | -0.029 |
| -> MBNMA |  | -0.102 | -0.179 | -0.027 |
| AE_1500 vs Placebo_0 | 0.117 |  |  |  |
| -> direct |  | -0.127 | -0.305 | 0.063 |
| -> indirect |  | -0.391 | -0.525 | -0.253 |
| -> MBNMA |  | -0.301 | -0.417 | -0.179 |
| AE_1250 vs Placebo_0 | 0.37 |  |  |  |
| -> direct |  | -0.129 | -0.462 | 0.219 |
| -> indirect |  | -0.259 | -0.35 | -0.16 |
| -> MBNMA |  | -0.251 | -0.348 | -0.149 |
| AE_1000 vs Placebo_0 | 0.029 |  |  |  |
| -> direct |  | -0.397 | -0.526 | -0.252 |
| -> indirect |  | -0.146 | -0.225 | -0.065 |
| -> MBNMA |  | -0.201 | -0.278 | -0.119 |
| AE_500 vs Placebo_0 | 0.067 |  |  |  |
| -> direct |  | -0.413 | -1.574 | 0.76 |
| -> indirect |  | -0.1 | -0.138 | -0.06 |
| -> MBNMA |  | -0.1 | -0.139 | -0.06 |
| AE_250 vs Placebo_0 | 0.061 |  |  |  |
| -> direct |  | 0.033 | -0.745 | 0.803 |
| -> indirect |  | -0.05 | -0.069 | -0.03 |
| -> MBNMA |  | -0.05 | -0.07 | -0.03 |

**Figure S5.** Node-splitting analysis (density plot). The value of title is the corresponding dose of that agent. CrI, credible interval.

## 7.2 Models’ selection

### 7.2.1 Non-linear functions and models fit comparison

A meta-analysis (i.e., a "split" NMA) of the different doses of physical activity as separate and unrelated treatments were performed. This step helps determine which function is more appropriate for the data and should be used in a model-based network meta-analysis (MBNMA) ^9^. **Figure S12** and **Figure S13** show the different responses of each dose to overall and different types of exercise, respectively (MD).

**Figure S6.**“Split” NMA of overall exercise.

**Figure S7.** “Split” NMA of different exercise agents.

### 7.2.2 Models selection

For our data, quadratic model shows the best fit and were therefore used in subsequent analyses.

**Table S8.** Models fit comparison

| **Model** | **DIC** | **SD** | **Deviance** | **Residual deviance** | **pD** |
| --- | --- | --- | --- | --- | --- |
| Emax (common treatment effects) | 34.7 | NA | -15.009 | 233.390 | 49.3 |
| Restricted cubic spline (common treatment effects; 3 knots) | -59.9 | NA | -118.259 | 130.140 | 58.3 |
| Restricted cubic spline (random treatment effects; 3 knots) | -85.2 | 0.116  (0.06,0.18) | -157.014 | 91.384 | 71.6 |
| Non-parametric monotonically up (common treatment effects) | 490.3 | NA | 443.485 | 691.884 | 46.5 |
| Quadratic (2^nd^ degree polynomial, common treatment effects) | 22.5 | NA | -30.805 | 217.594 | 53.1 |
| Quadratic (2^nd^ degree polynomial, random treatment effects) | -88.9 | 0.137  (0.09,0.20) | -159.674 | 88.725 | 71.1 |

In addition to the model fit index, a deviation plot showing the contribution of each data point to the residuals can also help to confirm the robustness of the model selection. The contribution of each data point to the posterior mean bias should be around 1, which indicates a good model fit ^10^. Deviation plots for overall **(Figure S14)** treatment effects **(Figure S15)** confirm the robustness of our model selection (i.e., deviations <1.5 except for a few data points in the overall exercise and RE at 750 METs-min, which are all below a contribution of 1.5).

**Figure S8.** Deviance plot at overall exercise

**Figure S9.** Deviance plots at treatment-level.

In addition, we also plotted the fit further to assess the degree of fit of the model. The fit values are plotted as connecting lines, and the observations in the original dataset are plotted as points. These plots can determine if the model fits the data well for different exercises and doses of the dose-response function^11^.

**Figure S10.** Fit plots at overall exercise level.

**Figure S11.** Fit plots at agent-level. CAE, continuous aerobic exercise. RE, resistance exercise. CE, combined aerobic and resistance exercise. TC, tai chi. HIIT, high-intensity interval training. CG, control group.

**Figure S12.** Prediction dose-response relationship at agent-level. PI, prediction interval. AE, continuous aerobic exercise. RT, resistance exercise. CE, combined aerobic and resistance exercise. HIIT, high-intensity interval training. CG, control group.

**Figure S13.** Treatment-level network. The first value indicates the specific intervention and the second one is the corresponding dose of that intervention.

Significant interval and 0-line intersection (light blue vertical dashed line): dose / identity / 95% CrI, Agent: exercise Significant interval Run 1: Start intersection: dose = 0; MD = 0; 95% CrI [0, 0]; SD = 0. End intersection: dose = 1500; MD = -0.193813; 95% CrI [-0.3402376, -0.04675285]; SD = 0.07486857, Inverted U-shape: Optimal dose point (red dot): dose / identity / 95% CrI / SD , Agent: exercise Best dose = 1100; MD = -0.2229843; 95% CrI [-0.2928639, -0.1532362]; SD = 0.0356193

**Figure S14.** Treatment-level network. The first value indicates the specific intervention and the second one is the corresponding dose of that intervention.

Agent: CE Significant interval Run 1: Start intersection: dose = 520; MD = -0.1496877; 95% CrI [-0.3072867, 0.0008437593]; SD = 0.0786047, End intersection: dose = 1200; MD = -0.2178917; 95% CrI [-0.4201096, -0.0147473]; SD = 0.1034087, Agent: HIIT Significant interval Run 1: Start intersection: dose = 1020; MD = -0.2239112; 95% CrI [-0.4533374, 0.001377533]; SD = 0.1159987, End intersection: dose = 1310; MD = -0.2618615; 95% CrI [-0.5279608, 0.0009242826]; SD = 0.1349197, Inverted U-shape: Optimal dose point (red dot): dose / identity / 95% CrI / SD , Agent: AE, Best dose = 980; MD = -0.2233799; 95% CrI [-0.2992831, -0.1422427]; SD = 0.04006132

**Table S9.** Predictions ranking (from best to worst)

| **Treatment** | **Mean** | **Median** | **2.50%** | **97.50%** |
| --- | --- | --- | --- | --- |
| HIIT_1200 | 10.55 | 8 | 2 | 29 |
| AE_1000 | 10.63 | 10 | 3 | 20 |
| HIIT_1300 | 10.66 | 7 | 2 | 33 |
| AE_830 | 11.29 | 11 | 2 | 22 |
| AE_1200 | 12.2 | 12 | 3 | 23 |
| HIIT_1000 | 12.23 | 10 | 1 | 30 |
| HIIT_1500 | 12.26 | 5 | 1 | 37 |
| CE_970 | 12.68 | 12 | 3 | 27 |
| CE_1100 | 12.7 | 11 | 2 | 30 |
| CE_1200 | 13.32 | 11 | 1 | 34 |
| CE_830 | 13.72 | 13 | 2 | 28 |
| AE_670 | 13.87 | 14 | 4 | 25 |
| AE_1300 | 14.08 | 14 | 2 | 28 |
| RT_1200 | 14.41 | 6 | 1 | 37 |
| HIIT_830 | 14.85 | 14 | 1 | 33 |
| RT_1100 | 14.9 | 8 | 2 | 36 |
| CE_690 | 15.79 | 16 | 2 | 30 |
| RT_970 | 15.89 | 13 | 3 | 35 |
| AE_500 | 17.78 | 18 | 7 | 28 |
| HIIT_670 | 17.83 | 18 | 1 | 35 |
| RT_830 | 17.85 | 18 | 4 | 34 |
| AE_1500 | 18.63 | 19 | 1 | 36 |
| CE_560 | 18.68 | 19 | 4 | 32 |
| RT_690 | 20.78 | 22 | 5 | 33 |
| HIIT_500 | 21.55 | 23 | 3 | 37 |
| CE_420 | 22.24 | 23 | 7 | 34 |
| AE_330 | 22.72 | 23 | 12 | 31 |
| RT_560 | 23.78 | 25 | 7 | 35 |
| HIIT_330 | 25.73 | 27 | 7 | 37 |
| CE_280 | 26.35 | 27 | 13 | 35 |
| RT_420 | 26.84 | 28 | 10 | 36 |
| AE_170 | 28.35 | 29 | 19 | 35 |
| RT_280 | 30 | 31 | 16 | 37 |
| HIIT_170 | 30.33 | 32 | 17 | 37 |
| CE_140 | 30.6 | 31 | 21 | 36 |
| RT_140 | 32.68 | 34 | 23 | 37 |
| Placebo_0 | 34.23 | 35 | 27 | 37 |

# Appendix 8.References

[1]Soori R, Rezaeian N, Khosravi N, et al. Effects of water-based endurance training, resistance training, and combined water and resistance training programs on visfatin and ICAM-1 levels in sedentary obese women[J]. Science & Sports, 2017, 32(3): 144-151.doi:10.1016/j.scispo.2016.12.004

[2]Yoshizawa M, Maeda S, Miyaki A, et al. Additive beneficial effects of lactotripeptides intake with regular exercise on endothelium-dependent dilatation in postmenopausal women. Am J Hypertens. 2010;23(4):368-372. doi:10.1038/ajh.2009.270

[3]Yoon JR, Ha GC, Ko KJ, Kang SJ. Effects of exercise type on estrogen, tumor markers, immune function, antioxidant function, and physical fitness in postmenopausal obese women. J Exerc Rehabil. 2018;14(6):1032-1040. Published 2018 Dec 27. doi:10.12965/jer.1836446.223

[4]Wen HJ, Huang TH, Li TL, Chong PN, Ang BS. Effects of short-term step aerobics exercise on bone metabolism and functional fitness in postmenopausal women with low bone mass. Osteoporos Int. 2017;28(2):539-547. doi:10.1007/s00198-016-3759-4

[5]Wooten JS, Phillips MD, Mitchell JB, et al. Resistance exercise and lipoproteins in postmenopausal women. Int J Sports Med. 2011;32(1):7-13. doi:10.1055/s-0030-1268008

[6]Wu J, Oka J, Higuchi M, et al. Cooperative effects of isoflavones and exercise on bone and lipid metabolism in postmenopausal Japanese women: a randomized placebo-controlled trial. Metabolism. 2006;55(4):423-433. doi:10.1016/j.metabol.2005.10.002

[7]Rossi FE, Diniz TA, Fortaleza ACS, et al. Concurrent Training Promoted Sustained Anti-atherogenic Benefits in the Fasting Plasma Triacylglycerolemia of Postmenopausal Women at 1-Year Follow-up. J Strength Cond Res. 2018;32(12):3564-3573. doi:10.1519/JSC.0000000000001732

[8]Lee JA, Kim JW, Kim DY. Effects of yoga exercise on serum adiponectin and metabolic syndrome factors in obese postmenopausal women. Menopause. 2012;19(3):296-301. doi:10.1097/gme.0b013e31822d59a2

[9]Tan S, Du P, Zhao W, Pang J, Wang J. Exercise Training at Maximal Fat Oxidation Intensity for Older Women with Type 2 Diabetes. Int J Sports Med. 2018;39(5):374-381. doi:10.1055/a-0573-1509

[10]Seo DY, Lee SR, Kim HK, et al. Independent beneficial effects of aged garlic extract intake with regular exercise on cardiovascular risk in postmenopausal women. Nutr Res Pract. 2012;6(3):226-231. doi:10.4162/nrp.2012.6.3.226

[11]Pereira R, Krustrup P, Castagna C, et al. Multicomponent recreational team handball training improves global health status in postmenopausal women at the long term - A randomised controlled trial. Eur J Sport Sci. 2023;23(8):1789-1799. doi:10.1080/17461391.2023.2184725

[12]Rossi FE, Fortaleza ACS, Neves LM, et al. Combined training (strength plus aerobic) potentiates a reduction in body fat but only functional training reduced low-density lipoprotein cholesterol in postmenopausal women with a similar training load. J Exerc Rehabil. 2017;13(3):322-329. Published 2017 Jun 30. doi:10.12965/jer.1734940.470

[13]Nazarabadi PN, Etemad Z, Hoseini R, Moradi F. Anti-Inflammatory Effects of a Period of Aerobic Training and Vitamin D Supplementation in Postmenopausal Women with Metabolic Syndrome. Int J Prev Med. 2022;13:60. Published 2022 Apr 8. doi:10.4103/ijpvm.IJPVM_312_20

[14]Rossi FE, Diniz TA, Neves LM, et al. The beneficial effects of aerobic and concurrent training on metabolic profile and body composition after detraining: a 1-year follow-up in postmenopausal women. Eur J Clin Nutr. 2017;71(5):638-645. doi:10.1038/ejcn.2016.263

[15]Pospieszna B, Karolkiewicz J, Tarnas J, Lewandowski J, Laurentowska M, Pilaczyńska-Szcześniak Ł. Influence of 12-week Nordic Walking training on biomarkers of endothelial function in healthy postmenopausal women. J Sports Med Phys Fitness. 2017;57(9):1178-1185. doi:10.23736/S0022-4707.16.06528-2

[16]Ready AE, Naimark B, Ducas J, et al. Influence of walking volume on health benefits in women post-menopause. Med Sci Sports Exerc. 1996;28(9):1097-1105. doi:10.1097/00005768-199609000-00004

[17]Nishida Y, Tanaka K, Hara M, et al. Effects of home-based bench step exercise on inflammatory cytokines and lipid profiles in elderly Japanese females: A randomized controlled trial. Arch Gerontol Geriatr. 2015;61(3):443-451. doi:10.1016/j.archger.2015.06.017

[18]Paolillo FR, Borghi-Silva A, Arena R, Parizotto NA, Kurachi C, Bagnato VS. Effects of phototherapy plus physical training on metabolic profile and quality of life in postmenopausal women. J Cosmet Laser Ther. 2017;19(6):364-372. doi:10.1080/14764172.2017.1326610

[19]Park SM, Kwak YS, Ji JG. The Effects of Combined Exercise on Health-Related Fitness, Endotoxin, and Immune Function of Postmenopausal Women with Abdominal Obesity. J Immunol Res. 2015;2015:830567. doi:10.1155/2015/830567

[20]Kazemi SS, Heidarianpour A, Shokri E. Effect of resistance training and high-intensity interval training on metabolic parameters and serum level of [21]Sirtuin1 in postmenopausal women with metabolic syndrome: a randomized controlled trial. Lipids Health Dis. 2023;22(1):177. Published 2023 Oct 19. doi:10.1186/s12944-023-01940-x

[22]Mohanka M, Irwin M, Heckbert SR, et al. Serum lipoproteins in overweight/obese postmenopausal women: a one-year exercise trial. Med Sci Sports Exerc. 2006;38(2):231-239. doi:10.1249/01.mss.0000184584.95000.e4

[23]Miyaki A, Maeda S, Choi Y, Akazawa N, Tanabe Y, Ajisaka R. Habitual aerobic exercise increases plasma pentraxin 3 levels in middle-aged and elderly women. Appl Physiol Nutr Metab. 2012;37(5):907-911. doi:10.1139/h2012-069

[24]Maesta N, Nahas EA, Nahas-Neto J, et al. Effects of soy protein and resistance exercise on body composition and blood lipids in postmenopausal women. Maturitas. 2007;56(4):350-358. doi:10.1016/j.maturitas.2006.10.001

[25]Lee YK, Cho SY, Roh HT. Effects of 16 Weeks of Taekwondo Training on the Cerebral Blood Flow Velocity, Circulating Neurotransmitters, and Subjective Well-Being of Obese Postmenopausal Women. Int J Environ Res Public Health. 2021;18(20):10789. Published 2021 Oct 14. doi:10.3390/ijerph182010789

[26]Lee J A, Kim J H, Kim J W, et al. Effects of aerobic exercise on serum blood lipids, leptin, ghrelin, and HOMA-IR factors in postmenopausal obese women[J]. 한국산학기술학회 논문지, 2017, 18(2): 549-558.doi:10.5762/KAIS.2017.18.2.549

[27]Keawtep P, Sungkarat S, Boripuntakul S, et al. Effects of combined dietary intervention and physical-cognitive exercise on cognitive function and cardiometabolic health of postmenopausal women with obesity: a randomized controlled trial. Int J Behav Nutr Phys Act. 2024;21(1):28. Published 2024 Mar 5. doi:10.1186/s12966-024-01580-z

[28]Buttelli ACK, Costa RR, Farinha JB, et al. Pilates training improves aerobic capacity, but not lipid or lipoprotein levels in elderly women with dyslipidemia: A controlled trial. J Bodyw Mov Ther. 2021;26:227-232. doi:10.1016/j.jbmt.2020.10.007

[29]Kim JW, Kim DY. Effects of aerobic exercise training on serum sex hormone binding globulin, body fat index, and metabolic syndrome factors in obese postmenopausal women. Metab Syndr Relat Disord. 2012;10(6):452-457. doi:10.1089/met.2012.0036

[30]Kheirat F, Merzouk H, Merzouk A S, et al. One year changes in biochemical and redox markers in training menopausal women with adherence to Mediterranean diet[J]. Science & Sports, 2018, 33(1): e25-e32.doi:10.1016/j.scispo.2017.07.013

[31]Kemmler W, Von Stengel S, Engelke K, Kalender WA. Exercise decreases the risk of metabolic syndrome in elderly females. Med Sci Sports Exerc. 2009;41(2):297-305. doi:10.1249/MSS.0b013e31818844b7

[32]Heli V, Ihab H, Kun H, Brad M, Jessica W, Vera N. Effects of exercise program on physiological functions in postmenopausal women with metabolic syndrome. Int J Gerontol. 2013;7(4):231-235. doi:10.1016/j.ijge.2013.05.002

[33]He H, Wang C, Chen X, et al. The effects of HIIT compared to MICT on endothelial function and hemodynamics in postmenopausal females. J Sci Med Sport. 2022;25(5):364-371. doi:10.1016/j.jsams.2022.01.007

[34]Ha MS, Yook JS, Lee M, et al. Exercise training and burdock root (Arctium lappa L.) extract independently improve abdominal obesity and sex hormones in elderly women with metabolic syndrome. Sci Rep. 2021;11(1):5175. Published 2021 Mar 4. doi:10.1038/s41598-021-84301-x

[35]Gómez-Tomás C, Chulvi-Medrano I, Carrasco JJ, Alakhdar Y. Effect of a 1-year elastic band resistance exercise program on cardiovascular risk profile in postmenopausal women. Menopause. 2018;25(9):1004-1010. doi:10.1097/GME.0000000000001113

[36]Frank LL, Sorensen BE, Yasui Y, et al. Effects of exercise on metabolic risk variables in overweight postmenopausal women: a randomized clinical trial. Obes Res. 2005;13(3):615-625. doi:10.1038/oby.2005.66

[37]Elliott KJ, Sale C, Cable NT. Effects of resistance training and detraining on muscle strength and blood lipid profiles in postmenopausal women. Br J Sports Med. 2002;36(5):340-344. doi:10.1136/bjsm.36.5.340

[38]Dupuit M, Rance M, Morel C, et al. Effect of Concurrent Training on Body Composition and Gut Microbiota in Postmenopausal Women with Overweight or Obesity. Med Sci Sports Exerc. 2022;54(3):517-529. doi:10.1249/MSS.0000000000002809

[39]Diniz T A, Fortaleza A C S, Rossi F E, et al. Short-term program of aerobic training prescribed using critical velocity is effective to improve metabolic profile in postmenopausal women[J]. Science & Sports, 2016, 31(2): 95-102.doi:10.1016/j.scispo.2015.03.006

[40]Colado JC, Triplett NT, Tella V, Saucedo P, Abellán J. Effects of aquatic resistance training on health and fitness in postmenopausal women. Eur J Appl Physiol. 2009;106(1):113-122. doi:10.1007/s00421-009-0996-7

[41]Binder EF, Birge SJ, Kohrt WM. Effects of endurance exercise and hormone replacement therapy on serum lipids in older women. J Am Geriatr Soc. 1996;44(3):231-236. doi:10.1111/j.1532-5415.1996.tb00907.x

[42]Chagas EFB, Bonfim MR, Turi BC, Brondino NCM, Monteiro HL. Effect of Moderate-Intensity Exercise on Inflammatory Markers Among Postmenopausal Women. J Phys Act Health. 2017;14(6):479-485. doi:10.1123/jpah.2016-0319

[43]Akazawa N, Choi Y, Miyaki A, et al. Effects of curcumin intake and aerobic exercise training on arterial compliance in postmenopausal women[J]. Artery research, 2013, 7(1): 67-72.doi:10.1016/j.artres.2012.09.003

[44]Arca EA, Martinelli B, Martin LC, Waisberg CB, Franco RJ. Aquatic exercise is as effective as dry land training to blood pressure reduction in postmenopausal hypertensive women. Physiother Res Int. 2014;19(2):93-98. doi:10.1002/pri.1565

[45]Akazawa N, Choi Y, Miyaki A, et al. Curcumin ingestion and exercise training improve vascular endothelial function in postmenopausal women. Nutr Res. 2012;32(10):795-799. doi:10.1016/j.nutres.2012.09.002

[46]Ağıl A, Abıke F, Daşkapan A, Alaca R, Tüzün H. Short-term exercise approaches on menopausal symptoms, psychological health, and quality of life in postmenopausal women. Obstet Gynecol Int. 2010;2010:274261. doi:10.1155/2010/274261

[47]Akazawa N, Choi Y, Miyaki A, et al. Aerobic exercise training increases cerebral blood flow in postmenopausal women[J]. Artery Research, 2012, 6(3): 124-129.doi:10.1016/j.artres.2012.05.00

[48]Huang Caihua, Gao Songling, Liu Libin, et al. Effects of 24-Week Exercise Training on Serum Leptin Level in Obese Postmenopausal Women and Its Relationship with Lipid Metabolism. China Sport Science and Technology, 2007, (05): 98-101. DOI: 10.16470/j.csst.2007.05.003.

[49]Chen Chunyang, Miao Jie, Ren Lei. Effects of 24-Week Physical Exercise on Serum Leptin Level and Lipid Metabolism in Obese Postmenopausal Women. Chinese Journal of Gerontology, 2014, 34(18): 5228-5229.

[50]Wang Guiran. Effects of Combined Exercise on Metabolic Syndrome Parameters in Postmenopausal Breast Cancer Patients. Chinese Journal of Gerontology, 2016, 36(10): 2408-2410.

[51]Huang Caihua, Chen Junqing, Chen Yiguang, et al. Effects of Exercise on Serum Adiponectin Level and Insulin Resistance in Postmenopausal Women with Type 2 Diabetes Mellitus. Journal of Tianjin University of Sport, 2009, 24(05): 415-419. DOI: 10.13297/j.cnki.issn1005-0000.2009.05.002.

[52]Deng Xianqiang, Dong Junqiu. Effects of Brisk Walking Exercise on Estrogen and Blood Lipid Levels in Postmenopausal Women. Journal of Xi'an Physical Education University, 2006, (04): 68-70+78. DOI: 10.16063/j.cnki.issn1001-747x.2006.04.021.
